# Combined Systems Approaches Reveal Highly Plastic Responses to Antimicrobial Peptide Challenge in *Escherichia coli*

**DOI:** 10.1371/journal.ppat.1004104

**Published:** 2014-05-01

**Authors:** Justyna Kozlowska, Louic S. Vermeer, Geraint B. Rogers, Nabila Rehnnuma, Sarah-Beth T. A. Amos, Garrit Koller, Michael McArthur, Kenneth D. Bruce, A. James Mason

**Affiliations:** 1 King's College London, Institute of Pharmaceutical Science, London, United Kingdom; 2 King's College London, King's College London Dental Institute at Guy's, King's and St. Thomas' Hospitals, London, United Kingdom; 3 Department of Molecular Microbiology, John Innes Centre, Norwich, United Kingdom; Oregon State University, United States of America

## Abstract

Obtaining an in-depth understanding of the arms races between peptides comprising the innate immune response and bacterial pathogens is of fundamental interest and will inform the development of new antibacterial therapeutics. We investigated whether a whole organism view of antimicrobial peptide (AMP) challenge on *Escherichia coli* would provide a suitably sophisticated bacterial perspective on AMP mechanism of action. Selecting structurally and physically related AMPs but with expected differences in bactericidal strategy, we monitored changes in bacterial metabolomes, morphological features and gene expression following AMP challenge at sub-lethal concentrations. For each technique, the vast majority of changes were specific to each AMP, with such a plastic response indicating *E. coli* is highly capable of discriminating between specific antibiotic challenges. Analysis of the ontological profiles generated from the transcriptomic analyses suggests this approach can accurately predict the antibacterial mode of action, providing a fresh, novel perspective for previous functional and biophysical studies.

## Author Summary

Antimicrobial peptides (AMP) are small proteins with often potent antibacterial activity found in a variety of organisms, including humans. Understanding how these antibiotics operate is challenging and often controversial since many studies have necessarily focussed on identifying a single major cause of bacterial cell death while, increasingly, others have cautioned that AMPs are likely to have access to multiple bactericidal features. Systems biology is an emerging field that comprises a series of techniques capable of giving a global view of how bacteria respond to external stimuli. Here we have monitored changes in gene expression and metabolism in bacteria that have been challenged with sub-lethal concentrations of four different AMPs. By understanding how bacteria respond to a threat we can reveal how the bacteria perceive the AMP to be operating. Our approach provides a sophisticated bacterial perspective of the mode of action of each AMP and reveals that the bacteria have a vast array of weapons that can be marshalled to deal with distinct AMP threats. Indeed, around a third (or even more) of the bacterial machinery might be useful in dealing with antibiotic challenges, highlighting why antibiotic resistance is such a persistent problem.

## Introduction

The isolation of cecropins [1], magainins [2] and defensins [3] from insects, amphibians and mammals in the late 1980's and early 1990's, highlighted the potential of host defence peptides as sources of novel antibiotics [4]. This novel antibiotic potential encouraged researchers to develop structure activity relationships for cationic antimicrobial peptides (AMPs), with the anionic bacterial plasma membrane the presumed site of action for bactericidal activity [5]. There is increasing evidence however that each AMP may indeed have multiple effects on a bacterial cell and hence may have multiple ways of killing microbial targets. AMPs may therefore function as “dirty drugs” with different bactericidal strategies possible for distinct bacterial species [4]–[7]. Indeed, the innate immune system may have selected AMPs that can exert their antimicrobial activity in multiple ways since this is less likely to lead to resistance developing as seen with classical antibiotics that have a single, high affinity target [6]. Our understanding of how AMPs function is therefore far from complete. Attempts to optimize AMP potency in the laboratory, that focus on only one possible bactericidal mechanism, ignore the possibilities offered by taking a holistic approach that can reveal the true source(s) of bactericidal potency along with a better understanding of bacterial counter-measures.

The full power of ‘omics based research tools has yet to be brought to bear in antibiotic research [8]. Nevertheless, important insights have emerged regarding the scope of bacterial responses by comparing challenges with distinct AMPs [8]. These studies have focussed on the Gram-positive bacterial species *Bacillus subtilis* [9], *Staphylococcus aureus* [10] and *Streptococcus pneumoniae* [11] and have demonstrated the existence of complex regulatory patterns in which several signal transduction pathways were induced. The transcriptional response of *Escherichia coli* to cecropin A, the proline rich Bac7(1-35) and novispirin G10 has been characterised in separate studies [12]–[14]. Recent work in our laboratory has focussed on trying to understand the relative difference in antibacterial potency of structurally related AMPs to Gram-negative bacteria such as *Escherichia coli* and *Pseudomonas aeruginosa* [15]–[18]. Here, AMPs with structural features thought to enhance antibacterial potency and reduce toxicity have been developed for use against more challenging pathogens [19], [20]. These peptides, including D-LAK120-AP13, have been developed based on an understanding of a variety of naturally occurring peptides including magainin 2, buforin II and pleurocidin. Pleurocidin is a 25 amino acid AMP found in the skin and gills of *Pleuronectes americanus*, the Winter Flounder. Despite resembling magainin 2 in terms of length, cationic charge, hydrophobicity and secondary structure in a range of membrane mimetic environments [18], pleurocidin is typically ten times more potent against Gram-negative species. Pleurocidin has been shown to be capable of acting on bacterial membranes [21], with pore forming activity, but has also been suggested to enter bacterial cells and interrupt protein synthesis [22]. We have therefore compared its effect on *E. coli* with magainin 2, which has been considered the archetypal pore forming AMP, and with buforin II which is proposed to enter bacteria to exert a bactericidal effect [23], [24].

Since these peptides act at widely differing effective concentrations we hypothesised that studying their effects at sub-lethal concentrations would provide a detailed overview of the mechanisms of action of each AMP. We therefore devised a method that could efficiently identify conditions where bacteria responded to AMP challenge without introducing possible, non-specific complications that might result from large scale cell death. We therefore used ^1^H high resolution magic angle spinning (HR-MAS) NMR to identify the lowest AMP concentration that elicited a response from metabolically active, challenged bacteria. A robust, cross-validated, multivariate analysis identified metabolites whose levels were altered in response to AMP challenge. These were used to classify the AMP according to the elicited response whilst providing a first indication of whether *E. coli* responded in a generic or specific manner to AMP challenge. Having identified sub-lethal conditions where a response was confirmed, electron microscopy and transcript profile analyses enabled a detailed description of the *E. coli* response to AMP challenge.

## Results

**Figure 1:**
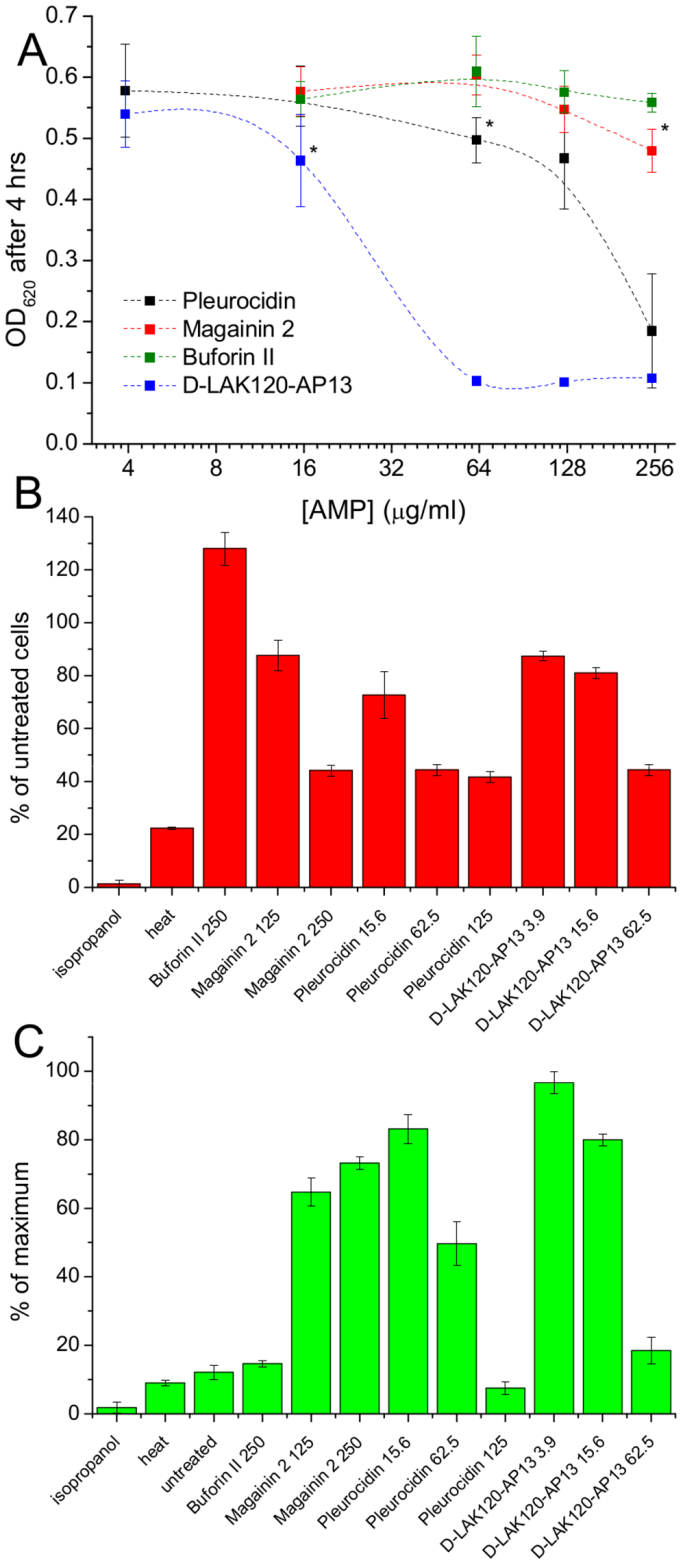
AMP challenge and multi-parameter assay of *E. coli* NCTC 9001. Overnight cultures were challenged with increasing amounts of each of four AMPs for 30°C (**A**). * indicates the peptide concentration causing a significant (*p*<0.1) reduction in OD_620_ relative to the lowest peptide concentration used. The membrane potential (**B**) of challenged bacteria as measured by the voltage sensitive dye DiBAC_4_ is expressed here as a percentage of the membrane potential determined for untreated cells. Esterase activity (**C**) determined by cleavage of 5,6-carboxyfluorescein diacetate expressed as a percentage of the maximum observed activity. Peptide concentrations are given in µg/ml.

**^1^H HR-MAS NMR metabolomics reveals threshold AMP concentration inducing a bacterial response** –The four cationic amphipathic AMPS selected to test the response of stationary phase *E. coli* (Table 1) were of similar length and were all C-terminally amidated with nominal charge ranging from +4 to +9. For the AMP challenge experiments presented here, higher bacterial cell densities (8×10^8^ CFU/ml) were required than is common in the broth microdilution assays [25] used to generate the MIC data (Table 1), in particular for HR-MAS analysis. At the higher bacterial titre, although the relative potency is similar, the effect of the four AMPs determined using such methods was somewhat different from the minimum inhibitory concentrations (MICs), with D-LAK120-AP13 having a substantially greater effect on bacterial numbers as detected in the challenge and recovery assay (Fig. 1A). Neither magainin 2 nor buforin II had a sufficiently inhibitory effect for a MIC to be determined at the higher titre. Nevertheless the amount of peptide causing a significant reduction in bacterial re-growth can be compared with D-LAK120AP13 effective at 15.6 µg/ml with substantially more pleurocidin (62.5 µg/ml) and magainin 2 (125 µg/ml) required for a significant effect. No effect on bacterial re-growth was observed for buforin II at any of the peptide concentrations tested (Fig. 1A). A multi-parameter assay was taken to assess the effect of peptide challenge on membrane potential (Fig. 1B), esterase activity (Fig. 1C) and membrane integrity in the challenged stationary phase bacteria and suggested that only the higher concentrations of pleurocidin and D-LAK120-AP13 were lethal. A dose dependent response to each of the four AMPs was observed but the membrane potential was not completely lost while the esterase activity was mostly higher than that observed in untreated cells; a hallmark of exposure to sublethal stress in *E. coli* [26].

**Table 1:** Sequences of peptides used in this study.

Peptide	Sequence	Length	Charge	Average Hydro-phobicity (*H*)*	Hydrophobic moment (µ*H*)	MIC (µg/ml) *E. coli* NCTC 9001	MIC (µg/ml) *P. aeruginosa* PAO1
Magainin 2	GIGKFLHSAKKFGKAFVGEIMNS	23	+4	−0.03	0.28	16.91±3.89	26.12±5.45
Pleurocidin	GWGSFFKKAAHVGKHVGKAALTHYL	25	+5	−0.02	0.22	1.79±0.60	4.47±2.36
Buforin II	TRSSRAGLQFPVGRVHRLLRK	21	+7	−0.37	0.30	>64.00	>64.00
D-LAK120-AP13	*KKLALALAKKWLPLAKKLALALAKK*	25	+9	−0.10	0.21	2.95±1.69	3.33±0.52

MIC data are as reported in (17) and (18).

*As determined by the Eisenberg (1982) Consensus scale (Ile, 0.73; Phe, 0.61; Val, 0.54; Leu, 0.53; Trp, 0.37; Met, 0.26; Ala, 0.25; Gly, 0.16; Cys, 0.04; Tyr, 0.02; Pro, −0.07; Thr, −0.18; Ser, −0.26; His, −0.40; Glu, −0.62; Asn, −0.64; Gln, −0.69; Asp, −0.72; Lys, −1.1; and Arg, −1.8).

**Figure 2:**
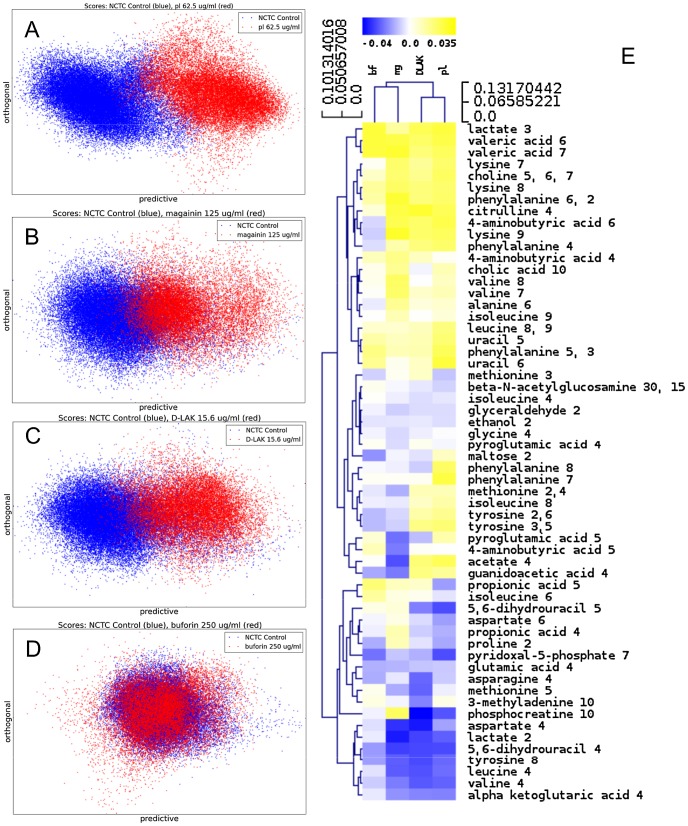
Metabolomic analysis by ^1^H HR-MAS NMR of lyophilised, stationary phase *E. coli* cell pellets. OPLS-DA scores plots are shown for challenge of *E. coli* NCTC 9001 at the following threshold concentrations; pleurocidin at 62.5 µg/ml (**A**), magainin 2 at 125 µg/ml (**B**), D-LAK120-AP13 at 15.6 µg/ml (**C**) and buforin II at 250 µg/ml (**D**). Hierachical clustered heatmap comparing loadings obtained from cross-validated OPLS-DA comparing untreated bacteria with AMP at the threshold concentrations indicated above (**E**).

One dimensional ^1^H NMR spectra were obtained for all samples. Principal component analysis identified outlier spectra resulting from either poor baseline or signal to noise, and either partial least squares (PLS) regression analysis or a series of orthogonal PLS-discriminant analysis (OPLS-DA) tests were used to interrogate the spectra. The latter was used in a step-wise manner to determine the lowest concentration of each AMP that caused a significant change in the spectra relative to spectra from untreated bacterial cell, as determined from Q^2^ (Table 2) where an arbitrary value ≥0.6 was taken to show a reliable model where the AMP challenge has a significant effect. This value can be compared with the value expected for a perfect separation of the two groups (Q^2^ = 1) and that obtained when the assigned classes (untreated or AMP challenged) are permutated as a means of representing no effect. 2D scores plots that resulted from each of the cross validated OPLS-DA analyses are shown in the supplementary material (Fig. S1 in File S1) while those for the threshold concentrations (data for the highest AMP concentration is shown for buforin II) are described here (Fig. 2A–D). A clear separation of the OPLS-DA scores was obtained at the indicated AMP concentration, identified as a threshold for a response detectable in the bacterial metabolomes. This threshold concentration varied considerably for the four AMPs and was directly related to the apparent antibacterial efficacy noted above. The threshold value, in turn, corresponded to a sub-lethal AMP concentration since bacterial growth remained greater than 50% of maximum (Fig. 1A), esterase activity was increased relative to untreated cells (Fig. 1C) while membrane potential was not completely lost (Fig. 1B). Hence the NMR metabolomic technique identified conditions where metabolically active *E. coli* were responding to the AMP challenge without simply reporting on bacterial cell death. Comparing the back-scaled loadings, each of the OPLS-DA comparisons between untreated bacteria and those challenged with each AMP, identified metabolites whose differing intensities correlated with the effect of each AMP. A hierarchical cluster analysis was used to reveal variation in metabolite levels (Fig. 2E). Both common and AMP specific variations in *E. coli* metabolite levels were observed in response to challenge with the four AMPs. Notably, the hierarchical analysis grouped the peptides according to their potency. Though not considering the magnitude or direction of changes in metabolite levels, network pathway analysis conducted using MetaboAnalyst [27], [28] matched pathways according to p-values obtained from pathway enrichment analysis and pathway impact from pathway topology (Fig. S2.1–S2.4 in File S1). Changes in alanine, aspartate and glutamate metabolism had the greatest impact and were a common feature of challenge with all four peptides with changes in pyruvate, butanoate and arginine/proline metabolism highlighted according to the distinct challenges.

**Table 2:** Predictive Q^2^ values for OPLS-DA models.

[AMP] (µg/ml)	Q^2^			
	Pleurocidin	Magainin 2	Buforin II	D-LAK120-AP13
3.9	0.32 (−0.29)	n.d	n.d.	0.37 (−0.30)
15.6	0.53 (−0.31)	0.29 (−0.36)	n.d.	0.59 (−0.28)*
62.5	0.81 (−0.41)*	0.20 (−0.31)	n.d.	0.81 (−0.31)
125	0.80 (−0.29)	0.68 (−0.34)*	n.d.	0.83 (−0.26)
250	n.d.	n.d.	−0.30 (−0.39)*	n.d.

Q^2^ values for cross validation performed with permutated classes are provided in parentheses.

* Key minimum concentrations.

The dynamic response of *E. coli* NCTC9001 to challenge with pleurocidin or magainin 2 was assessed over a period of 2 hours at the following intervals: 5 minutes, 15 minutes, 60 minutes and 120 minutes. The OPLS-DA scores plots (Fig. S3 in File S1) and corresponding Q^2^ (Table S1 in File S1) indicate that a response to AMP challenge at the level of the metabolome can be detected throughout the period tested. However, when the back-scaled loadings were compared in a hierarchical cluster analysis (Fig. S4 in File S1), modest but notable differences in the affected metabolites were discerned. This suggested that the bacterial response detected beyond an hour after challenge is characteristically distinct from that probed within the first 30 minutes. These conditions – 30 minutes incubation at the determined threshold concentration - were therefore used for subsequent electron microscopic and transcript profiling analyses of samples prepared in parallel to those used above.

### Scanning and transmission electron microscopy identifies differences in the response to each AMP

**Figure 3:**
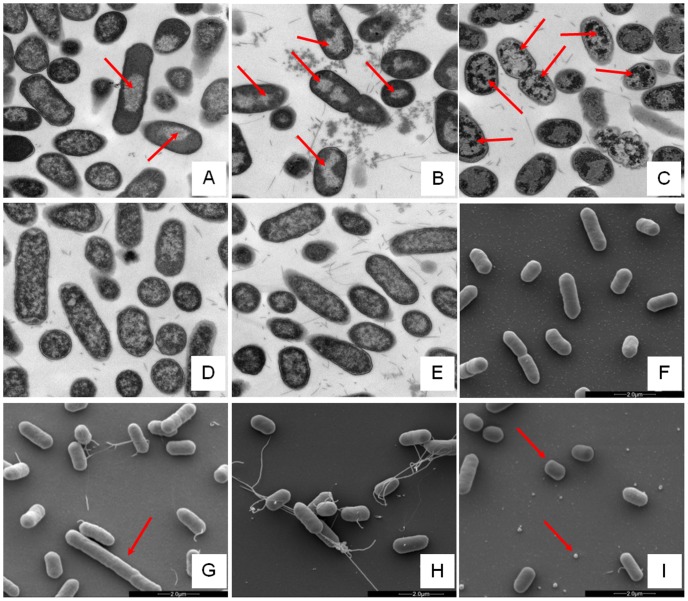
Electron microscopic analysis of *E. coli* response to AMP challenge. Transmission (**A–E**) and scanning (**F–I**) electron micrographs at ×25,000 magnification of either untreated (**E/F**) or AMP challenged *E. coli* NCTC 9001. Stationary phase bacteria were challenged for 30 minutes with AMPs above the threshold concentration that elicits a bacterial response as determined by the ^1^H NMR metabolomic study; 250 µg/ml magainin 2 (**A/G**), 125 µg/ml pleurocidin (**B/H**), 62.5 µg/ml D-LAK120-AP13 (**C/I**) and 250 µg/ml buforin II (**D**). Red arrows indicate features described in the results.

Changes in *E. coli* internal or external morphology in response to challenge with AMP were monitored respectively using transmission and scanning electron microscopy (TEM/SEM) at either one or four times the sub-inhibitory AMP threshold concentration known to induce a metabolomic response (Fig. 3; Fig. S5.1–5.10 in File S1). The bacterial response to each AMP challenge varied considerably and was in qualitative agreement with the metabolomic study; buforin II had no noticeable effect when compared with untreated bacterial cell controls (Fig. 3D–F; Fig. S5.2/5.9/5.10 in File S1), with each of the three other AMPS inducing substantial changes to external and/or internal morphologies. For magainin 2, a regular, almost circular nucleoid condensation was observed in some, but not all, cells (Fig. 3A; Fig. S5.8 in File S1) while some impairment of cell division was evident with extended rods observed (Fig. 3G). Pleurocidin also induced nucleoid condensation but this was much more widespread; observed throughout the bacterial cell population (Fig. S4.5/S4.6 in File S1). This was accompanied by some possible protein aggregation and the production of large amounts of a fibrous material (Fig. 3B). In addition to the production of the fibrous material, SEM indentified moderate vesicle production, a known envelope stress response in Gram-negative bacteria [29]. Finally, D-LAK120-AP13 induced dramatic changes in both the internal (Fig. 3C) and external *E. coli* morphologies (Fig. 3I). Extensive release of outer membrane vesicles was evident which was coincident with a loss of the normal rod shape, consistent with bacteria budding prematurely (Fig. 3I). Inside bacterial cells, extensive nucleoid condensation and protein aggregation was observed throughout the bacterial cell population (Fig. 3C; Fig. S5.3/S5.4 in File S1). Taken together, although there were some qualitative similarities in the response of *E. coli* cells to each of the three more potent AMPs, markedly distinct responses to each peptide were observed overall. Transmission electron micrographs obtained at higher magnification and with AMP added at a concentration above the detected threshold value indicated that, for all four peptides, the bacterial envelope remained intact and no release of cell contents was apparent (Fig. S5.1 in File S1).

### Global transcriptome response identifies some generic, but largely AMP specific, responses

**Figure 4:**
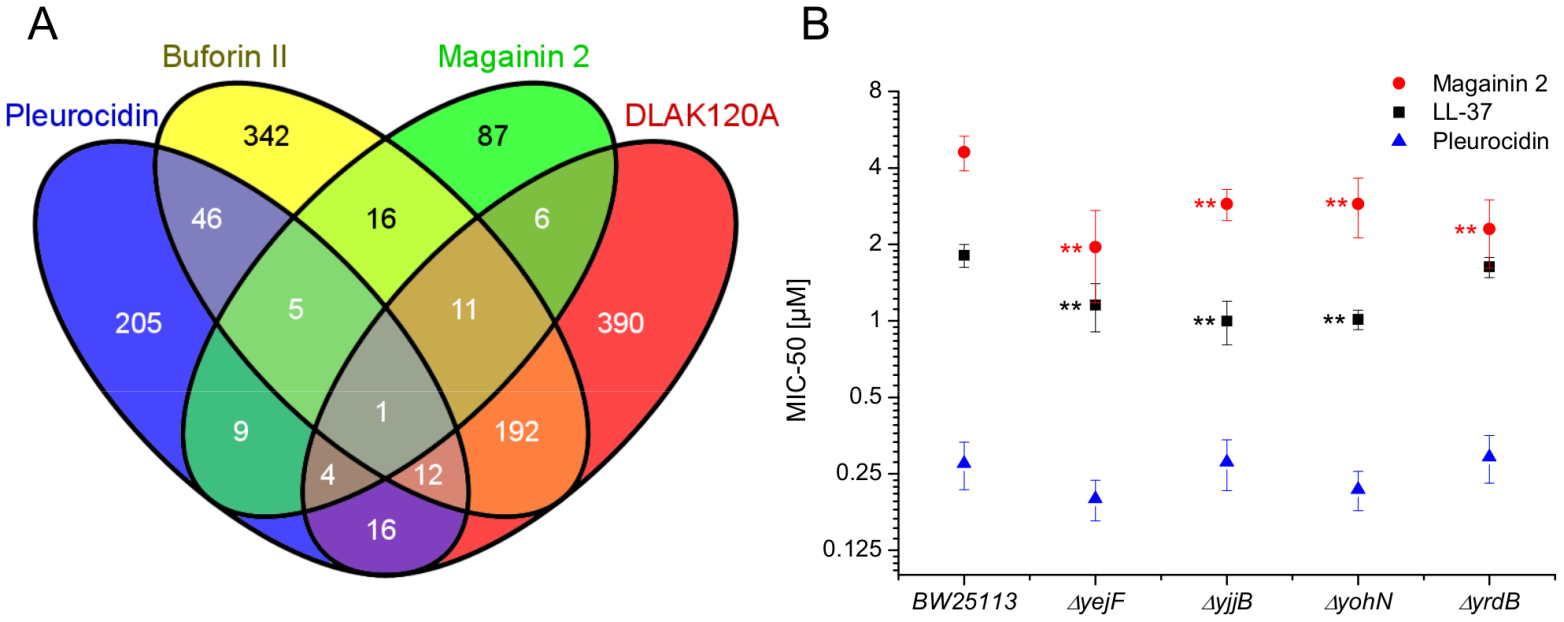
Transcript profiles and role of individual genes in response to AMP challenge. Four way Venn diagram (**A**) showing the distribution of differentially expressed genes detected by the GeneChip *E. coli* Genome 2.0 Array (*p*≤0.05) following challenge of stationary phase *E. coli* NCTC 9001 with each of four AMPs at sub-inhibitory concentrations known to elicit a bacterial response; pleurocidin at 62.5 µg/ml, magainin 2 at 125 µg/ml, D-LAK120-AP13 at 15.6 µg/ml and buforin II at 250 µg/ml. The entries in the Venn correspond to the number of affected genes. Effect on sensitivity of *E. coli* BW25113 to magainin 2, pleurocidin and LL-37 of mutations in four of six genes commonly regulated in response to AMPs of natural origin (**B**).

The response of *E. coli* to challenge with the four AMPs was then probed at the level of the transcriptome. Transcript profile changes in the NCTC 9001 strain, a clinical isolate from a patient with cystitis with cystitis, were monitored using the *E. coli* Genome 2.0 Array where four strains including laboratory, uropathogenic and enteropathogenic strains are featured. Due to the high degree of similarity between strains, in the majority of cases, a single probe set represents the equivalent ortholog in all four strains. All genes that are subsequently described in detail are found in both laboratory (K12 substr. MG1655) and uropathogenic (CFT073) strains with the majority also found in the two enteropathogenic strains. Principal component analysis of the twenty most differentially expressed genes across all groups showed the three independent replicates of each condition clustered together indicating the AMP challenge and transcript profiling assay were reproducible (Fig. S6 in File S1). Further analysis, where either an arbitrary significance level (*p*≤0.05) for differential gene expression or manual manipulation of significance levels leading to an optimal separation by principal components, generated lists of differentially expressed genes related to each treatment. *E. coli* genomes commonly encode between approximately 4,200 and 5,500 protein coding genes [30], [31]. Of the approximately 10,000 probe positions, between 139 and 632 differentially expressed unique genes (*p*≤0.05) were detected for each treatment following challenge with AMP at the threshold concentration eliciting a bacterial response. This corresponds to 2.5–15.0% of the available genome. Magainin 2 induced differential expression of only 139 genes which contrasted with the much greater number of genes whose expression was altered in response to challenge with either buforin II or D-LAK120-AP13; 625 and 632 respectively. Pleurocidin induced differential expression of 298 genes. The distribution of differentially expressed genes according to each AMP treatment is represented in a Venn diagram and reveals that the vast majority (76.3%) are specific to each of the four AMP challenges (Fig. 4A). Only 32 differentially expressed genes, 2.4% of the total, were common to at least three treatments while there was only one, *yjjB*, which was common to all four treatments. Qualitatively therefore, transcriptomic data supported the electron microscopy findings as, while common responses can be identified, the dominant impression was of a largely specific response to each AMP challenge

Mapping those discriminating metabolite changes with most impact (Fig. S2.1–S2.4 in File S1) onto their respective Kyoto Encyclopaedia of Genes and Genomes (KEGG) pathways identified differentially expressed genes with a potentially key role in mediating the response to AMP challenge. Changes in alanine, aspartate and glutamate metabolism were common to all four peptides and changes in expression of *gltX*, *dapA* and *metB*, coding for respectively glutamyl-tRNA synthetase, dihydropicolinate synthase and cystathionine gamma-synthase, were observed in the gene lists though these did not always satisfy the significance thresholds used above. Knockout mutants of *dapA* and *gltX* are not available from the Keio collection but Δ*metB* and five other knockout mutants (Δ*cyoA*, Δ*cyoC*, Δ*cyoD*, Δ*speB*, and Δ*argR* coding respectively for cytochrome o uniquinol oxidase subunits II, III and IV, agmatinase and arginine repressor), linked to changes in arginine/proline metabolism, were tested for altered sensitivity to AMP challenge though none was found.

Up-regulated in response to challenge by all four AMPs, *yjjB*, encodes a 157 amino acid, conserved, inner membrane protein predicted to have four trans-membrane helices but with no known function. Of the five genes whose expression was generically affected by the three AMPs of natural origin, three were up-regulated in response to AMP challenge; *manA* codes for mannose-6-phosphate isomerise, *cysE* codes for a serine acetyltransferase and *yohN* codes for a 112 amino acid integral membrane protein annotated and established as a periplasmic modulator of nickel and cobalt efflux and renamed *rcnB* [32]. In contrast, *yejF*, part of an ABC transporter identified as a possible nickel, and probable microcin C transporter [33], and *yrdB*, which codes for a highly anionic, glutamine rich, 85 amino acid hypothetical protein from the DUF1488 superfamily, are down-regulated. Comparison of the growth of parent strain BW25113 and four knockout mutants (Δ*yejF*, Δ*yjjB*, Δ*yohN* and Δ*yrdB*) obtained from the Keio collection [34] confirmed *yohN* confers sensitivity to Co^2+^ and possibly Ni^2+^ (Fig. S7 in File S1). The growth of these strains was also tested in the presence of AMPs (Fig. 4B). While the MIC for pleurocidin was not affected by the presence of any of the four deletions, a modest but significant (*p*<0.05) increase in sensitivity was observed for all four deletion strains when challenged by magainin 2. When the experiment was repeated with LL-37, an AMP of human origin, three of the deletions rendered the bacteria more sensitive while deletion of *yrdB* had no effect.

**Figure 5:**
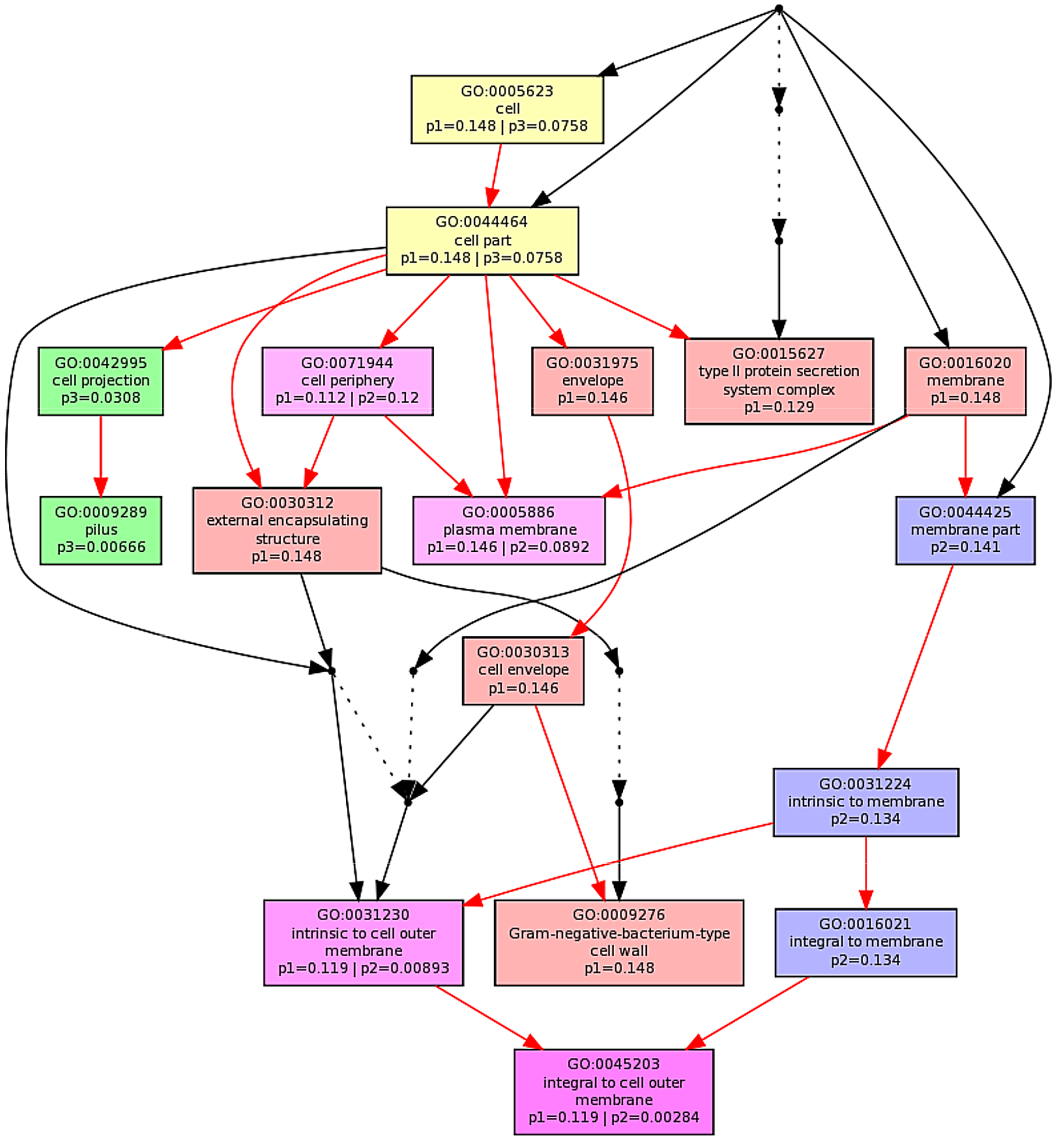
Multi GOEAST comparison of gene ontology (GO) terms relating to cellular component for differential gene responses in stationary phase *E. coli* NCTC 9001. Challenge was induced with sub-inhibitory concentrations of pleurocidin (red: p1), magainin 2 (blue: p2) and buforin II (green: p3). Red arrows represent relationships between two enriched GO terms, black arrows between enriched and un-enriched terms and black dashed arrows represent relationships between two un-enriched GO terms. Raw *p* values for GO terms have been adjusted using the Benjamini-Hochberg method allowing FDR<15%.

The ontological profile related to each challenge offers another view of how closely related the response to each AMP is to each other. Here, instead of comparing individual genes on the basis of their identity, the comparison is based on the cellular component, biological process or molecular function and is less affected by redundancy or more subtle changes in response and consequently better reflects the fundamentals of the bacterial response. Ontological analysis, which employed a Benjamini-Hochberg method to control false discovery rate (FDR) and displays statistically overrepresented, differentially expressed genes in a graphical format according to their relationships in a hierarchical tree, was carried out on gene lists comprising the 200–250 most differentially expressed genes for each of the individual AMP treatments (Fig. S8–S13 in File S1) and for comparisons of up to three AMP treatments (Fig. 5; Fig. S8/S9 in File S1). The three AMPs derived from natural sources are suspected of acting on different cellular components. Indeed, comparing gene ontology (GO) term enrichment for cellular components (Fig. 5) showed a very different profile for each of magainin 2, buforin II and pleurocidin. Magainin 2 appears confined to affecting membrane components (Fig. 5; Fig. S10 in File S1) and had little effect on molecular functions or biological processes. Buforin II, in contrast, did not impact on any membrane components, instead focussing on components in the “cell” or “cell part” (Fig. 5; Fig. S11A in File S1) where 41% of the differentially expressed genes related to binding are found in the analysis of molecular function (Fig. S11B in File S1). Pleurocidin elicited responses both in membrane components and in the cell itself (Fig. 5; Fig. S12 in File S1) with biological processes, in particular polysaccharide and macromolecule metabolism and transport, impacted. This was reinforced by the finding that some 35 genes related to transporter activity were differentially expressed (Fig. S13 in File S1). These observations reinforce the view that AMPs impact on bacterial cells in distinct and AMP-specific ways. When the top 250 genes differentially expressed in response to challenge with D-LAK120-AP13 were analyzed, very few enriched pathways were found when biological processes were considered, with no enriched cellular components or molecular function identified. This indicates a non-specific response for this designed peptide notwithstanding its shared responses with buforin II observed above.

## Discussion

### The value of a combined approach

When taken together, the metabolomic, electron microscopy and transcript profiling analyses reveal a combination of generic and specific responses to challenge with AMPs that share many physicochemical features but that differ in their modes of action. All four peptides used were cationic, of similar lengths, and will adopt conformations with secondary amphipathicity in the supposed target of the *E. coli* inner membrane. For the analytical techniques used, some strengths and weaknesses were identified, so underscoring the value of a combined approach. The electron micrographs provided compelling evidence of AMPs induction of manifestly different responses in *E. coli* challenged at both inhibitory and sub-inhibitory concentrations. The images however provide only circumstantial evidence as to the mechanism of action of each peptide. Instead, quantitative information or details of the molecular mechanisms involved are needed to pinpoint how each peptide operates. Transcript profiling provides a rich vein of information on the bacterial response. The individual gene products implicated have suggested a wide range of experiments that will illuminate further how bacteria attempt to fight off challenges posed by AMPs. Transcript profiling may also be more sensitive than the other approaches used since it alone was able to identify a significant response to buforin II which, even when administered at 250 µg/ml did not cause any perceived effect on either the internal or external cellular morphology or register a response as detected by ^1^H HR-MAS NMR. The transcript profiling method remains expensive however and the consumable costs per sample make its use in a high throughput manner unattractive. The NMR metabolomic technique has the advantage of having low per sample consumable costs which enables a much greater range of test conditions to be assessed. NMR metabolomics is also highly reproducible and provides quantitative information on this greater number of test conditions. It would therefore be attractive to consider whether it could be used as a standalone method for interrogating bacterial responses to challenge. In the present study however, while both generic and specific changes in metabolites were identified in response to AMP challenge, generic changes may appear overestimated when compared with the information provided by transcript profiling or micrographs. This may be due to common metabolic pathways underpinning a series of distinct bacterial responses and a much larger scale investigation, with a larger panel of both distinct and more closely related AMPs is now warranted. This would allow greater weight to be afforded to certain key metabolites, known to be altered in response to a given class of AMP with known influence on bacterial stress responses.

### Life and death at the membrane?

This study investigates whether studying bacterial responses, when challenged with carefully defined sub-lethal concentrations of antibiotic, provides a detailed systems wide view of the mechanism of action. The mechanism of action of cationic amphipathic helix forming antimicrobial peptides has received considerable attention in the past two decades with much work focussed on the pore forming activity of magainin 2 and related peptides [35]. Considered an archetypal pore forming peptide, there is nevertheless evidence that for at least one microbial target, *Saccharomyces cerevisiae*, magainin 2 can enter the cell and interfere with DNA integrity [36] while pore forming activity that causes graded dye release is linked to a mechanism that involves translocation of the peptide across the membrane [37]. Finally, MD simulations have shown that magainin-H2, when forming a disordered toroidal pore does indeed translocate to the internal leaflet of the membrane [38]. Set against these studies are a range of data on the structurally and physico-chemically related, but considerably more potent, pleurocidin which is known to have pore forming activity [21] but is also capable of entering bacteria to interfere with the synthesis of macromolecules [22]. We have recently solved the high resolution structures of both magainin 2 and pleurocidin in the anionic detergent SDS (PDB entries 2LSA and 2LS9 respectively) and found similar regions of flexibility around the glycine residues in the middle section of the sequence (Gly 13/18 – magainin 2; Gly 13/17 – pleurocidin). Only in the membranes that most closely mimic the inner membrane of Gram-negative bacteria are any differences between the two peptides observed; here pleurocidin adopts a notably more disordered conformation under these conditions [18]. The more disordered conformation of pleurocidin in the *E. coli* target membrane may be related to possible pore formation [39] or the proposed intracellular targeting strategy [22] which, in both cases, would serve to boost its potency.

Previous ‘omics based studies comparing AMPs action in Gram-positive bacterial species found that there was very little overlap in response between *Streptococcus pneumoniae* that had been challenged with each of three rather different antimicrobial peptides [11], while two earlier studies [9], [10], which focussed on peptides with the plasma membrane as a presumed common target, found rather more overlap. We therefore decided to test whether a more holistic approach would succeed in discriminating between the different modes of actions of magainin 2 and pleurocidin and place their differing membrane activities in a wider context, enabling a more sophisticated understanding of their respective mechanisms of action while explaining the greater potency of pleurocidin. In the present study, the combined approach was readily capable of distinguishing pleurocidin and magainin 2 on the basis of the bacterial responses observed in their metabolomic and transcript profiles with electron micrographs bringing these differences into sharp relief. Despite the shared physicochemical properties and conformational propensities of the two peptides and presumed initial target of the bacterial inner membrane, transcript profiling identified only 19 genes whose differential expression was common to both AMP challenges, with differential expression of some 399 genes being a specific response to either pleurocidin or magainin 2. The *E. coli* response to AMP challenge is therefore highly adaptable and is most sensitive to the differing bactericidal strategies of each peptide. Large scale changes in the internal morphology of *E. coli*, following challenge with sub-inhibitory concentrations of each AMP, provides circumstantial evidence that both magainin 2 and pleurocidin can enter Gram-negative bacteria, with the more profound effects of pleurocidin suggesting a greater proficiency. Improvements in imaging technologies and labelling techniques may open the way, in future, for the more precise localisation of both peptides but it is apparent that a simple description of AMP bactericidal mechanisms that rests solely on studying the membrane interaction in isolation is inadequate. This is particularly relevant for the goal of increasing potency.

We have also studied the structural properties of buforin II which is considered to operate via an intracellular targeting strategy [18]. Buforin II has a greater affinity for nucleic acids, has a greater nominal charge at +7 and is less hydrophobic. The proline kink in buforin II is known to be crucial for enabling translocation into the *E. coli* cytosol [24]. Notably, in all membranes that we have studied, the peptide adopts an extended helical conformation, rather than one rich in α-helix, and has only barely detectable antibacterial activity against planktonic *E. coli* cultures [18]. We therefore included buforin II in the present study since we hypothesised that the bacterial response to this peptide would highlight responses to pleurocidin that are related to an intracellular targeting strategy. Neither the NMR metabolomic nor electron micrograph studies though identified a strong response to even very elevated concentrations of this peptide; consistent with our previous work which identified only a very weak effect against planktonic cultures of either *E. coli* or *P. aeruginosa* [18]. Nevertheless, a large number of significantly differentially expressed genes in response to buforin II challenge were detected by transcript profiling. While around 64 differentially expressed genes were detected in common to challenge with buforin II and pleurocidin, 33 differentially expressed genes were common to buforin II and magainin 2 with a further 534 differentially expressed genes identified that were not affected by either magainin 2 or pleurocidin. Only six differentially expressed genes were identified as a common response to these three AMPs. This further emphasises the plasticity of the *E. coli* response and indicates that bacteria have a large repertoire of responses to challenges.

Considering the ontology of the differentially expressed genes can suggest how each individual AMP operates but, when used in comparison, as here, the relative importance of the properties of each AMP is revealed and supported the view that these three peptides adopt distinct bactericidal strategies. The ontological profiles reveal near orthogonal changes in transcript profiles following sub-lethal challenge with the three different AMPs of natural origin. Comparison of GO terms with existing paradigms for the mode of action of each AMP supports the view that the present, combined approach faithfully reveals the mechanism of action, notwithstanding the extra detail that identifies a range of effects that may contribute to bacterial cell death. In particular, the identification of eight GO terms linked to membranes supports the established view that magainin 2 largely acts on the plasma membrane of Gram-negative bacteria. In contrast, within the top 200 differentially expressed genes, no membrane GO terms were linked to the action of buforin II which is considered to seek intracellular targets. This is further supported by the distribution of GO terms since the effect on binding and a host of biosynthetic pathways is acute. For pleurocidin, where multiple bactericidal mechanisms have been proposed, there is substantial overlap between the cellular component GO terms with those affected by magainin 2. This indicates that the bacterial membrane is indeed a common target. However, in contrast with magainin 2, pleurocidin impacts on a large number of intracellular biological process, in particular macromolecule metabolic and transport processes. This strongly indicates a multifaceted antibacterial strategy underpins the high antibacterial potency of this AMP.

### Can understanding the bacterial response be exploited to improve AMP potency?

The high plasticity of the bacterial response to AMP challenge suggests that deletion of one gene is unlikely to have a great impact on sensitivity. This view is supported by the study of mutants identified by mapping metabolite changes with the greatest pathway impact onto their respective pathways and further work will be required to more effectively disrupt such pathways in order to identify any relationship with sensitivity to AMPs.

Nevertheless, six gene products were identified that were significantly and uniformly affected by the three AMPs derived from natural sources. Of these six genes, two were down-regulated; *yrdB* an anionic 85 amino acid hypothetical protein and *yejF*. The *yejF* gene codes for the ATPase in the ABC transporter YejABEF which, when mutated, confers resistance to microcin C [33]. The speculated role of YejABEF as a nickel transporter has been questioned as it is phylogenetically distant from other oligopeptide transporters [33]. However, since *yejF* is down-regulated in the present study in response to all three peptides obtained from natural sources and its deletion renders *E. coli* more sensitive to both magainin 2 and LL-37, this behaviour does support the earlier finding that the activity of this protein can have a considerable effect on peptide antibiotic potency. Indeed, while mutations in *yejABEF* confer resistance to microcin C in *E. coli*, deletion of *yejF* in *Salmonella enterica* increased sensitivity to AMPs, including both human beta defensins 1 and 2 (hBD-1 and hBD-2) [40].

Of the four genes that are up-regulated, *cysE* and *manA* are widely distributed amongst taxa, including animals, making them less attractive as an antibiotic target. In contrast, with a distribution that is concentrated in *Enterobacteriaceae* and with yet to be tested functions, *yohN and yjjB* might be more attractive targets for further investigation and possible targets for adjuvants that could boost the potency of the host innate immune response. Deletion of these genes caused a significant but only modest increase in sensitivity to magainin 2 and LL-37 while the potency of pleurocidin was unaffected. These results show that the combined systems approach is indeed capable of identifying genes that regulate resistance/sensitivity in *E. coli* but that the large number of potentially differentially expressed genes at the disposal of such bacteria will mitigate the effect that silencing one gene product may have.

Finally, we were interested to contrast the expected results for the three peptides representing naturally occurring AMPs with the bacterial response to a peptide, D-LAK120-AP13, which was composed of D-amino acids only. D-LAK120-AP13 was designed in an attempt to circumvent the effect of proteases secreted by target pathogens, and incorporate structural features, including high cationicity and propensity for adopting α-helix rich conformation [41] - and hence inserting into and disordering the *E. coli* inner membrane - and a proline kink, affording conformational flexibility [20] that facilitates penetration into bacteria [23], [24]. The robust and potent effect of this peptide against *E. coli* was evident with a significant metabolomic response even at very low peptide concentrations. Circumstantial evidence for the ability to penetrate within bacterial cells was shown by transmission electron microscopy, with the most profound changes due to challenge with any of the four AMPs observed, and transcript profiling. Again underlining the plasticity of the *E. coli* response, transcript profiling identifies a further 390 differentially expressed genes that were uniquely affected by D-LAK120-AP13 although, interestingly, there is considerable degree of overlap with the response to buforin II with 192 differentially expressed genes in common. These two peptides have a greater nominal cationic charge in solution at neutral pH than either pleurocidin or magainin 2 and both incorporate a proline induced kink in the secondary amphipathic conformation. Taken together, the data support highly effective entry of D-LAK120-AP13 into Gram-negative bacterial cells and it is this that may underpin its high antibacterial potency.

With four distinct but physicochemically related AMPs now tested by an integrated systems biology approach, a total of at least 1342 differentially expressed genes (*p*≤0.05) have been identified as being potential tools that can be manipulated by the bacteria to overcome AMP challenge. This is equivalent to between 24 and 32% of the total *E. coli* genome and suggests, with more structurally diverse AMPs yet to be tested, that bacteria have a wide variety of means of overcoming AMP challenges. Understanding these responses enables both the mode of action of AMPs to be elucidated as well as suggesting strategies to overcome these defences. The approach may find generic applicability in the study of antibiotic-bacteria arms races.

## Materials and Methods

### Materials

The peptides (Table 1) were all amidated at the C-terminus and were purchased from Pepceuticals Ltd (Nottingham, UK) as desalted grade or synthesised in house (D-LAK120-AP13) and were further purified using water/acetonitrile gradients using a Waters SymmetryPrep C8, 7 µm, 19×300 mm column.

### Bacterial culture and challenge

Cultures of *Escherichia coli* NCTC 9001, a strain isolated from a patient with cystitis, were grown overnight in Mueller-Hinton broth (MH) at 37°C. Once the OD_620_ reached ≈1.0, 1 ml aliquots of bacterial suspension were transferred into 1.5 ml microcentrifuge tubes and aqueous solutions of peptides - magainin 2, buforin II, pleurocidin and D-LAK120-AP13 were added at the following concentrations: 250 µg/ml, 125 µg/ml, 62.5 µg/ml, 15.6 µg/ml, 3.9 µg/ml and incubated for 30 min at 37°C. In order to be able to monitor the microbial recovery and growth, 10 µl of each suspension was sampled in 190 µl fresh medium onto a 96-well microplate. The OD_620_ was measured at time 0 and after 4 h of incubation at 37°C. The microcentrifuge tubes were centrifuged at 5000× *g* for 5 min and the bacterial pellets were snap frozen in liquid nitrogen, lyophilised and kept at −20°C until further use. Pellets from triplicate tubes were combined for subsequent HR-MAS analysis. Each challenge was independently repeated nine times.

### HR-MAS NMR

High-resolution magic angle spinning (HR-MAS) experiments were performed on a Bruker Avance 400 MHz spectrometer equipped with a 4 mm ^1^H/^13^C HR-MAS probe. The lyophilised cell pellets were thawed at room temperature, transferred to an NMR rotor inserts and rehydrated with 30 µl of D_2_O 2 hours before the acquisition. 1D spectra were recorded at a constant temperature of 310 K with magic angle spinning applied at 5 kHz. 1D ^1^H spectra were recorded using a standard cpmgpr1d spin echo pulse (cpmgpr; Bruker) with water presaturation during recycle delay of 1 second and a total of 128 scans were acquired. The spectral width was 16.02 ppm and ^1^H 90 pulse length was 7.81 µsec. The free induction decay was multiplied with an exponential function corresponding to a line broadening of 0.3 Hz. Phase correction was performed manually and automatic baseline correction was applied. A total of 120 samples were analysed with between 6 and 13 samples per treated condition and 17 control samples (no AMP treatment). A number of 2D experiments were run to facilitate identification of the compounds: homonuclear J-resolved 2D correlation with presaturation during relaxation delay using gradients (J-Res; jresgpprqf), ^1^H/^13^C correlation via direct inept transfer, phase sensitive using states, with decoupling during acquisition (HSQC 13C; AA-hsqcwg-13C), 2D homonuclear shift correlation with presaturation during relaxation delay (COSY; cosyprqf) all acquired using standard Bruker pulse sequences. Spectra were Fourier transformed, manually phase and automatically baseline corrected and calibrated with 2,2,3,3-D4-3-(Trimethylsilyl) propionic acid sodium salt (TMSP-2,2,3,3-D4) with reference signal at 0 ppm.

### Assignment

Resonances were assigned based on *J*-couplings partners revealed by COSY, multiplicities derived from J-Res, statistical correlation spectroscopy (STOCSY) [42] and both ^1^H and ^13^C chemical shifts with reference to the *E. coli* metabolome database [43].

### Multivariate data analysis

Spectra were analysed by principal component analysis (PCA) and orthogonal partial least squares discriminant analysis (OPLS-DA) using software developed in our laboratory for a previous study [44] incorporating the nonlinear iterative partial least squares (NIPALS) algorithm [45]. First, the spectra were aligned to the reference peak and spectral regions such as water and reference peak (4.8 ppm and 0 ppm, respectively) and regions of no interest and/or no spectral information were removed. Spectra were then normalised using probabilistic quotient normalization (PQN) [46] and autoscaled but not bucketed. Cross-validation was performed where 66% of the samples were used as a training set and the remaining 33% as a test set, ensuring that the number of samples in the test set was proportional to the total number of samples from each class, and that at least one sample from each class was present in the test set. To choose the number of components for the model, a leave-one-out cross-validation was carried out on the samples in the training set, and the F1-score used to choose the number of components, with the additional constraint to use a maximum of 10 components. This double cross-validation was repeated 2000 times with randomly chosen samples in the training and test set to prevent bias due to the choice of training or test set. This leads to 3×2000 models (in the supplementary information, each of these models leads to a point on the scores plot, but loadings and weights are presented as averages over all these models). Finally, this procedure was repeated with randomly generated class assignments to provide a reference value for Q^2^. The chosen number of components minus one was then used as an OPLS filter and a PLS-DA analysis with two components was carried out on the filtered data to yield one predictive and one orthogonal component. The Q^2^ value was calculated as Q^2^ = 1−(PRESS/TSS) where PRESS is the sum of squared differences between the known and predicted classes, and TSS is the sum of squared differences between the known classes and their average ( = the total variance). Q^2^ thus gives a measure of the goodness of fit after cross validation, and although it is generally considered to be “good” when its value is higher than 0.5 [47], [48] we have compared it to a reference value by computing Q^2^ for models where the classes were assigned randomly [47], [48]. In each case, genuine or permutated class assignments, the Q^2^ value quoted is the mean of all models. Back-scaled loadings plots [49] were used to identify resonances with high variance and high weight, therefore the discriminating resonances, and verified against the peak intensity of the original spectra after PQN normalisation. Freely available MultiExperiment Viewer (MeV) which is a part of the TM4 Microarray Software Suite [50] was used for hierarchical cluster (HCL) analysis and generation of heatmaps. Euclidian distance algorithm was used to compute the differences between two gene expression levels (metabolite level changes) and the average linkage method was used to define the distances.

### Scanning and transmission electron microscopy

Both SEM and TEM were used to examine the structural changes in bacteria induced by AMPs. Samples for the imaging were prepared in parallel with the samples used for HR-MAS NMR and hence represent bacteria in stationary phase. For SEM, the pellet obtained after centrifugation was fixed in 25 µl of 2.5% (v/v) glutaraldehyde in 0.2 M sodium cacodylate buffer and kept at 4°C until further use. In 24-well tissue culture plates 20 µl aliquots of vortexed bacterial pellet was smeared on 12 mm round poly-L-lysine (BD Biosciences, Bedford) cover slips with adjacent chambers filled with sufficient amount of 0.2 M sodium cacodylate to prevent drying of the slides and kept in a hydration chamber for 2 h. Cover slips were then washed with 0.2 M sodium cacodylate buffer followed by rinsing with 30%, 70%, 100%, 100%, and 100% ethanol and incubating for 10 min between each wash. Hexamethyldisilazane (HMDS) was used for drying of the specimen by washing cover slips in 50/50 100% ethanol/HMDS for 10 min followed by the final wash in HMDS for 10 min. The coverslips with dehydrated cells were mounted on the specimen stubs and sputter coated with gold. Micrographs were acquired with FEI Quanta 200F FEG scanning electron microscope. Bacterial pellets for TEM processing were prepared as described above. Cells were pelleted by centrifugation and the pellet was post fixed in 1% osmium tetroxide in 0.1 M phosphate buffer for 60 min an RT. The pellet was dehydrated by exposure to a graded series of ethanol (10%, 70% for 10 min each) followed by four washes in 100% ethanol for 15 min each. Next, the pellet was subjected to two washes in propylene oxide, 10 min each. Tubes containing pellets were constantly rotated during the washes and the following procedures and the washes were performed in the fume hood. The supernatant was removed and the pellet placed into a mixture of 50% resin and propylene oxide for 90 min and transferred to 100% resin overnight before polymerisation at 60°C for 24 hours. The resin blocks were sectioned with Leica Ultra-cut ultramicrotome to semi-thick sections (0.75 µm–2 µm) and stained with toluidine blue and used to determine the areas for thin sectioning (90 nm). The sections were then placed onto 150 mesh copper grids coated with pioloform support film. Grids were then stained with uranyl acetate and lead citrate before viewing on Hitachi H7600 transmission electron microscope. For both techniques, around 15 images were taken for each treatment. The following magnifications were used and images were selected that are representative of the effect observed: 700×, 5000×, 12000×, 25000×, 70000×.

### GeneChips

GeneChip experiments were performed using the Affymetrix (Santa Clara, CA) *E. coli* Genome 2.0 Array with effective, response inducing, sub-MIC AMP concentrations determined from the HR-MAS metabolomic study; pleurocidin 62.5 µg/ml, buforin II 250 µg/ml, magainin 2 125 µg/ml and D-LAK120-AP13 15.6 µg/ml. Each array includes approximately 10,000 probe sets for all 20,366 genes present in four strains of *E. coli* over the entire open reading frame (ORF); K12 (MG1655 laboratory strain), CFT073 (uropathogenic), 0157:H7-EDL953 (enteropathogenic) and O157:H7-Sakai (enteropathogenic). RNA was extracted using RiboPure and enriched using MICROB*Express* Bacterial mRNA Enrichment Kit after the DNA digestion step (Life Technologies, Paisley, UK) At each step the quality of RNA was assessed using Pico100 (Picodrop Ltd, Hinxton, UK). cDNA was synthesized from mRNA and purified using Qiagen MinElute PCR (Qiagen, Manchester, UK). cDNA was then fragmented and labeled using terminal transferase and biotinylated Affymerix GeneChip labelling reagent according to the manufacturer's instructions. Fragmentation and labeling were assessed with the 2100 Bioanalyzer (Agilent Technologies, Wokingham, UK) to obtain the size distribution and yield. cDNA was kept at −80°C until microarray hybridization. Hybridization of the target to the GeneChip was prepared according the standard Prokaryotic Target Hybridisation protocol according to the manufacturer's instructions. The efficiency of the hybridization step was assessed by examining hybridization of Poly-A controls provided for the Affymetrix GeneChip. Arrays were scanned on an Affymetrix GCS3000 microarray system and image acquisition, quantification and data analysis were performed using Affymetrix Command and Expression Console Software. Data were normalized using the Robust Multi-array Average (RMA) algorithm built into Expression Console. Pre-selection of gene lists for each treatment was performed using Qlucore Omics Explorer (Qlucore AB, Lund, Sweden). First, ANOVA across all samples identified the twenty most differentially expressed genes according to each replicated treatment. These were then assessed by principal component analysis (Fig. S6 in File S1) to confirm that independently replicated experiments produced consistent results. Signal intensities for gene expression were then averaged across technical duplicates/triplicates and log transformed. For the gene annotation enrichment analysis, differentially expressed genes in treatment versus control samples were selected by a paired, homoscedastic t-test with a significance cutoff of *p*<0.05 and lists for the four AMP treatments were then compared using Venny [51]. Microarray data are available in the ArrayExpress database (www.ebi.ac.uk/arrayexpress) under accession number E-MTAB-1703. To better understand the differences between the effects of the four treatments, significance thresholds that identified the approximate top 200–250 differentially expressed genes were selected; *p*≤0.0184 for buforin II and D-LAK120-AP13, *p*≤0.0425 for pleurocidin and *p*≤0.078 for magainin 2. These lists were analyzed using the GOEAST Gene Ontology Enrichment Analysis Software Toolkit where the Benjamini-Hochberg option was selected allowing an FDR up to 15% [52]. Discriminating metabolite changes, identified from HR-MAS NMR, were then mapped onto the KEGG pathway using BioCyc Omics Data Analysis [53] and genes related to given metabolic pathway checked against consistently differentially expressed genes, whether or not they had passed the significance test described above.

### Multiparameter viability assays

In order to assess the functionality and cellular integrity of bacteria we used the following viability assays: membrane potential assay, esterase activity assay and BacLight Live-Dead stain for microscopy [26].

As previously, *E. coli* NCTC 9001 were grown from glycerol stocks in Muller-Hinton broth overnight at 37°C without shaking until an OD_620_ of 1.0 was reached. 1 ml aliquots of culture were challenged for 30 min with four peptides at and below the threshold concentrations established with NMR. Cells were then harvested by centrifugation at 5,000× g for 5 min and washed in 50 mM phosphate buffer (pH 7.0). For BacLight Live/Dead stain cells were diluted to 4×10^8^ CFU/ml, whereas for the remaining assays cells were diluted to 2×10^8^ CFU/ml. All experiments were performed at room temperature. Negative controls were obtained either by treatment with 70% isopropanol for 10 min and removed by centrifugation at 5,000× g for 5 min and re-suspension in PBS, or by heat killing at 85°C for 10 min on a heat block. Assays were performed in black, flat bottom, 96-well plates and read on a Synergy HT multi-mode microplate reader (BioTek, Winooski, VT)

### Membrane potential

25 mg of dye DiBAC_4_ (Anaspec, Fremont, CA) was reconstituted in 2.42 ml ethanol to obtain a 20 mM stock solution which was stored at −20°C. The stock was diluted further with water to working concentration of 12.5 µM immediately before use. 20 µl of 12.5 µM dye was added to a 96-well plate, covered by 180 µl bacterial suspension in PBS and mixed. The plate was incubated in the dark for 5 minutes and fluorescence emission was measured (excitation 485 nm, emission 535 nm). Since membrane damage leads to higher fluorescence intensity, values were background corrected and expressed as a reciprocal before being normalised with untreated cells defined as being 100% and isopropanol treated cells defined as 0%.

### Esterase activity

5 mg of esterase substrate 5,6-carboxyfluorescein diacetate (CFDA) was dissolved in 1.086 ml dimethyl sulfoxide (DMSO) to obtain 10 mM stock kept at −20°C. Stock was diluted 40× in water immediately before use to obtain working concentration of 250 µM, which was pre-aliquoted to a 96-well plate. 180 µl of bacterial suspension in PBS was added to the plate and mixed with the detection solution. The plate was incubated in dark for 30 minutes with occasional shaking and fluorescence emission measured (excitation 485 nm, emission 535 nm).

### LIVE/DEAD BacLight

LIVE/DEAD BacLight kit (Life Technologies, Paisley, UK) was used to measure membrane integrity. Harvested cells were reconstituted with saline and 3 µl of the dye mixture (1.5 µl of SYTO9 (3.34 mM) and 1.5 µl of propidium iodine (20 mM)) was added to each 1 ml of bacterial suspension and mixed. Tubes were incubated for 15 minutes in the dark with occasional shaking and fixed with 20% paraformaldehyde (PFA) and kept at 4°C. Specimens were viewed on an Olympus BX60 microscope fitted with an Andor Ultrahigh-resolution CCD setup. A ×20 oil immersion lens was used to obtain a 200 µm field width. Excitation and emission filters were 480/520 nm and 515/560 nm respectively.

### MIC testing

Parent strain BW25113 and Keio knockout strains [34] for Δ*yejF*, Δ*yjjB*, Δ*yohN*, and Δ*yrdB* were obtained from the Coli Genetic Stock Center (Yale University, New Haven, CT). The activities of the peptides were assessed in planktonic suspension in polypropylene 96 well plates (Greiner Bio-one, Frickhausen, Germany) according to a modified broth dilution assay (54). Bacteria were grown without shaking in 50 ml Mueller-Hinton (MH) broth at 37°C. Peptides (pleurocidin, magainin 2 and LL-37) were tested in duplicates with two rows allocated for each peptide. In each of columns 2–11, 50 µl of MH broth was added under sterile conditions. In the first column of each row, 50 µl of 256 µg/ml stock peptide solutions, prepared in distilled water, were added and then the broth from the second column was pipetted into the first column and thoroughly mixed before being deposited again in the second column. This process was repeated throughout the tray providing a twofold dilution of peptide with each row. Bacteria with an A_620_ of 0.001 were then added to each well in volumes of 50 µl giving a further twofold dilution and a final volume of 100 µl per well. The final column was used either as sterility control (100 µl broth) or negative control (no peptide). Plates were incubated overnight at 37°C and the A_620_ read. Growth curves prepared from duplicates were fitted to determine the peptide concentration required to inhibit growth by 50% (MIC_50_). The MIC_50_ quoted for each peptide (Fig. 4) is an average value from at least two independent repeats.

## Supporting Information

File S1 **Supplementary figures and table.**
(PDF)
