# Supplementary material for: Combined Systems Approaches Reveal Highly Plastic Responses to Antimicrobial Peptide Challenge in Escherichia coli
Source: PLoS Pathog. 2014 May 1;10(5):e1004104. doi: 10.1371/journal.ppat.1004104 (PMC4006907; doi:10.1371/journal.ppat.1004104)
Supplement: File S1 — Supplementary figures and table. (PDF) [file ppat.1004104.s001.pdf]

Supplementary Figures

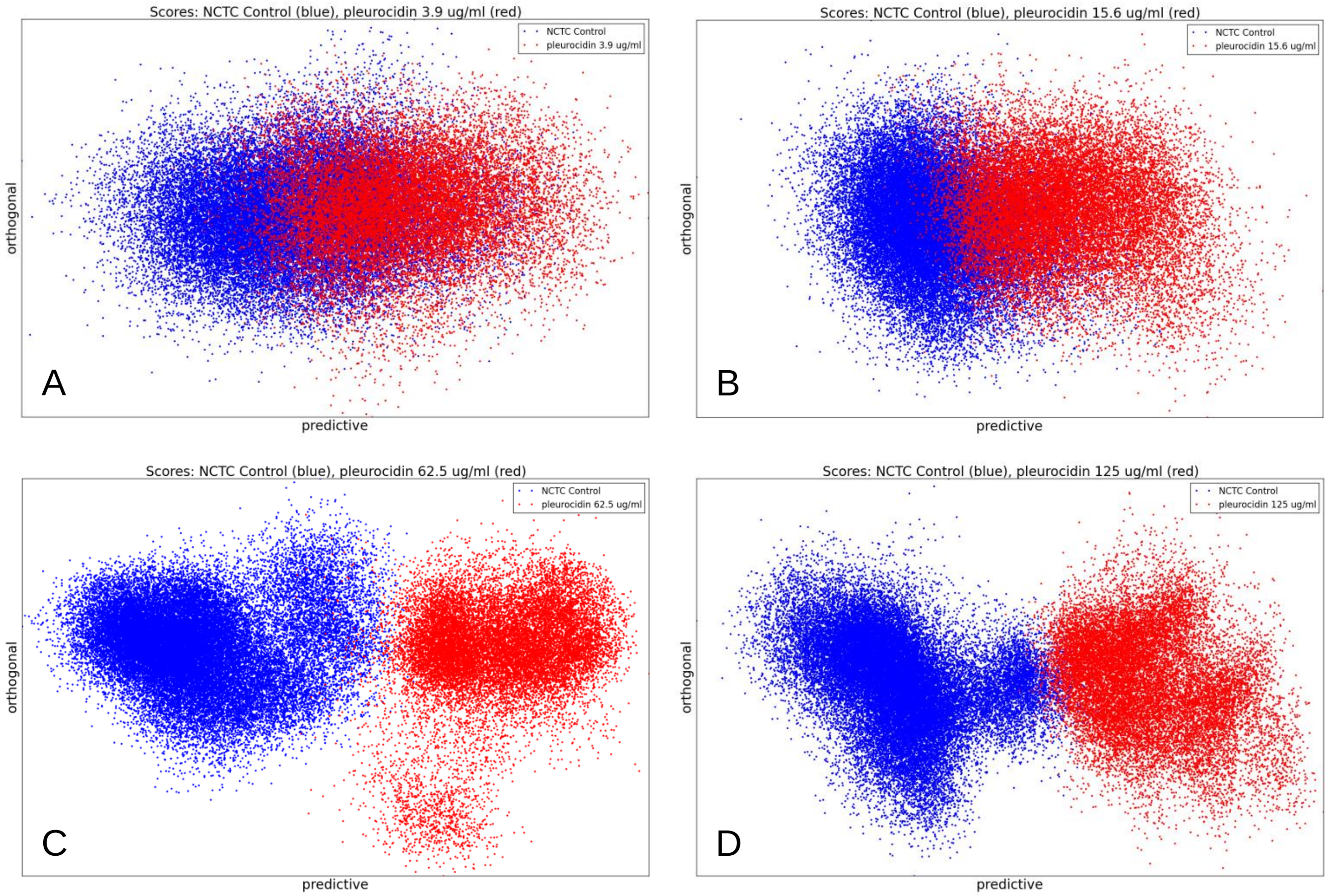

Supplementary Figure 1.1. OPLS-DA scores for comparisons of ^{1}H HR-MAS NMR spectra of control *E. coli* NCTC 9001 and those challenged with pleurocidin at 3.9 µg/ml (**A**), 15.6 µg/ml (**B**), 62.5 µg/ml (**C**) and 125 µg/ml (**D**). In all panels blue dots represent scores from unchallenged bacteria while red dots represent scores from the respective treatments.

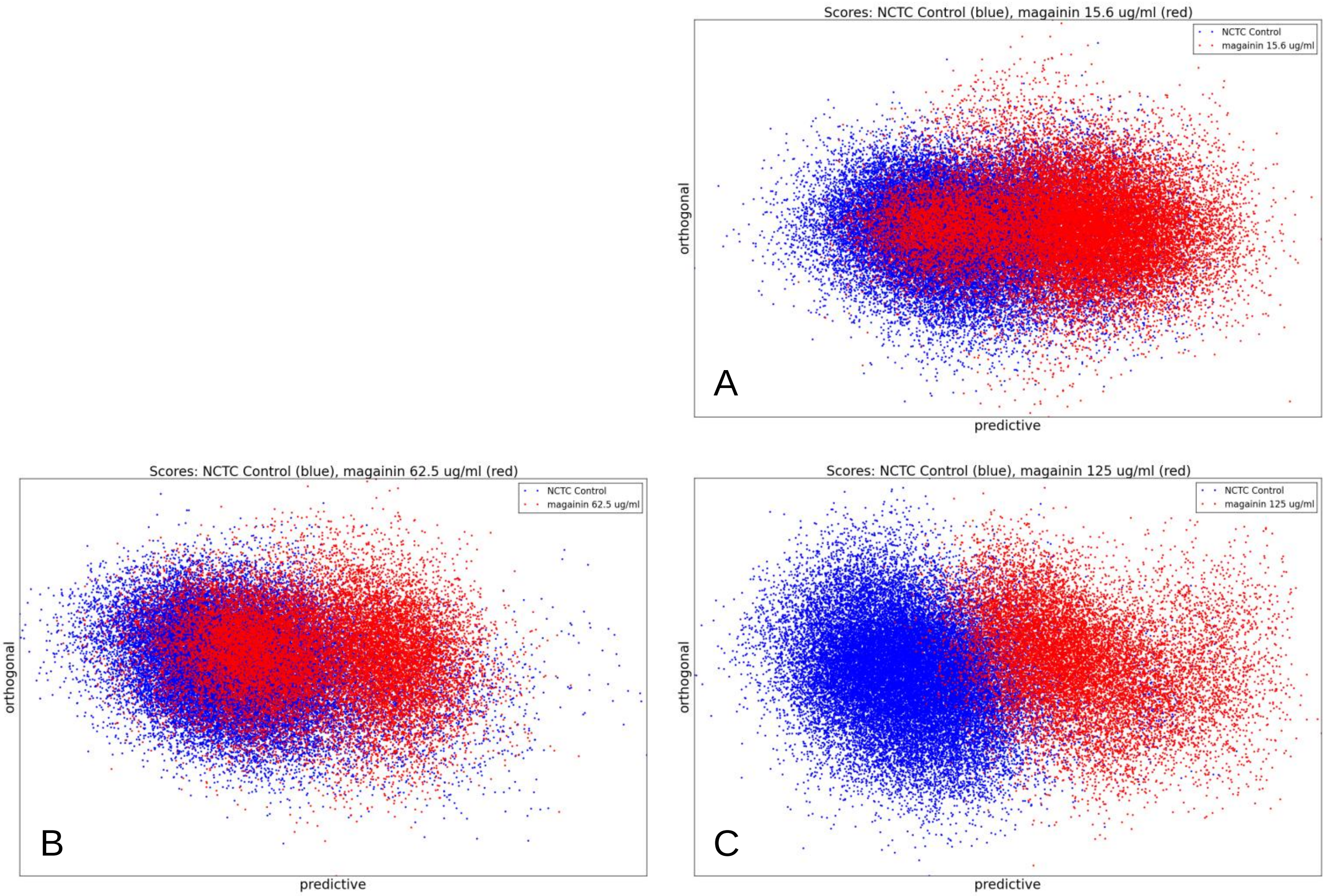

Supplementary Figure 1.2. OPLS-DA scores for comparisons of ^{1}H HR-MAS NMR spectra of control *E. coli* NCTC 9001 and those challenged with magainin 2 at 15.6 µg/ml (**A**), 62.5 µg/ml (**B**) and 125 µg/ml (**C**). In all panels blue dots represent scores from unchallenged bacteria while red dots represent scores from the respective treatments.

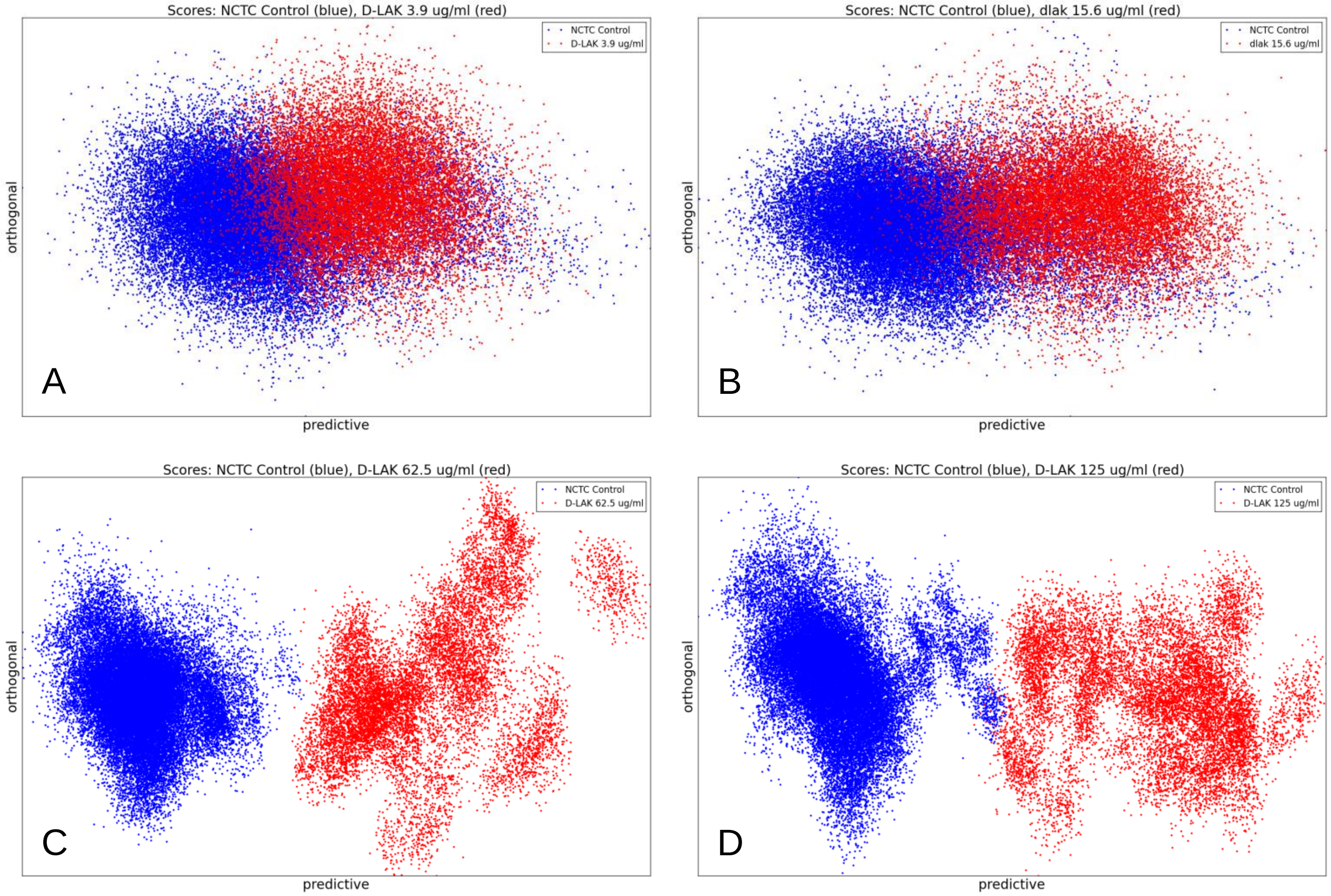

Supplementary Figure 1.3. OPLS-DA scores for comparisons of ^{1}H HR-MAS NMR spectra of control *E. coli* NCTC 9001 and those challenged with D-LAK120-AP13 at 3.9 µg/ml (**A**), 15.6 µg/ml (**B**), 62.5 µg/ml (**C**) and 125 µg/ml (**D**). In all panels blue dots represent scores from unchallenged bacteria while red dots represent scores from the respective treatments.

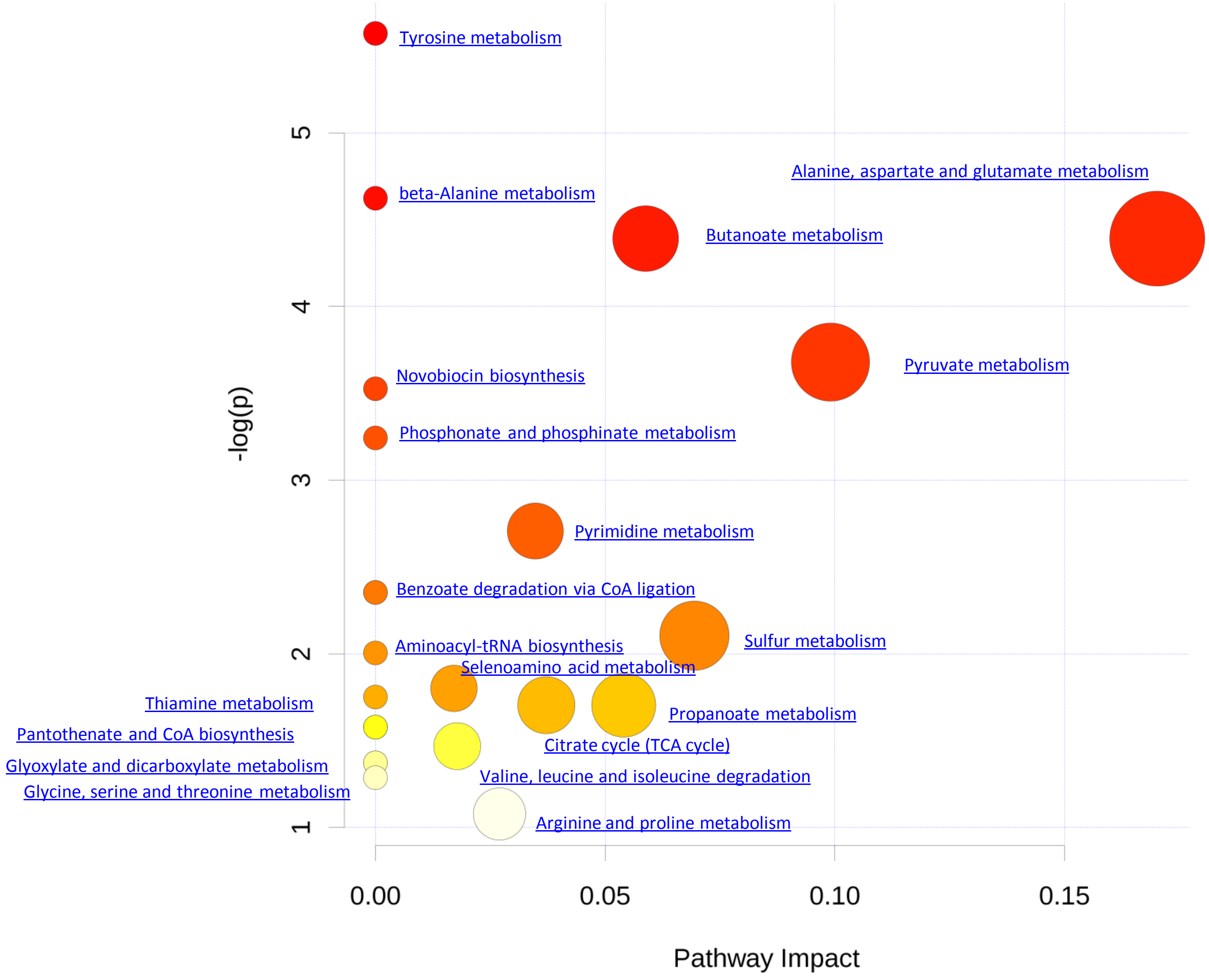

Supplementary Figure 2.1. Network pathway analysis by MetaboAnalyst software showing matched pathways according to p-values from pathway enrichment analysis and pathway impact values from pathway topology analysis based on the identified NMR resonances distinguishing control from the treatment with pleurocidin (62.5 µg/ml).

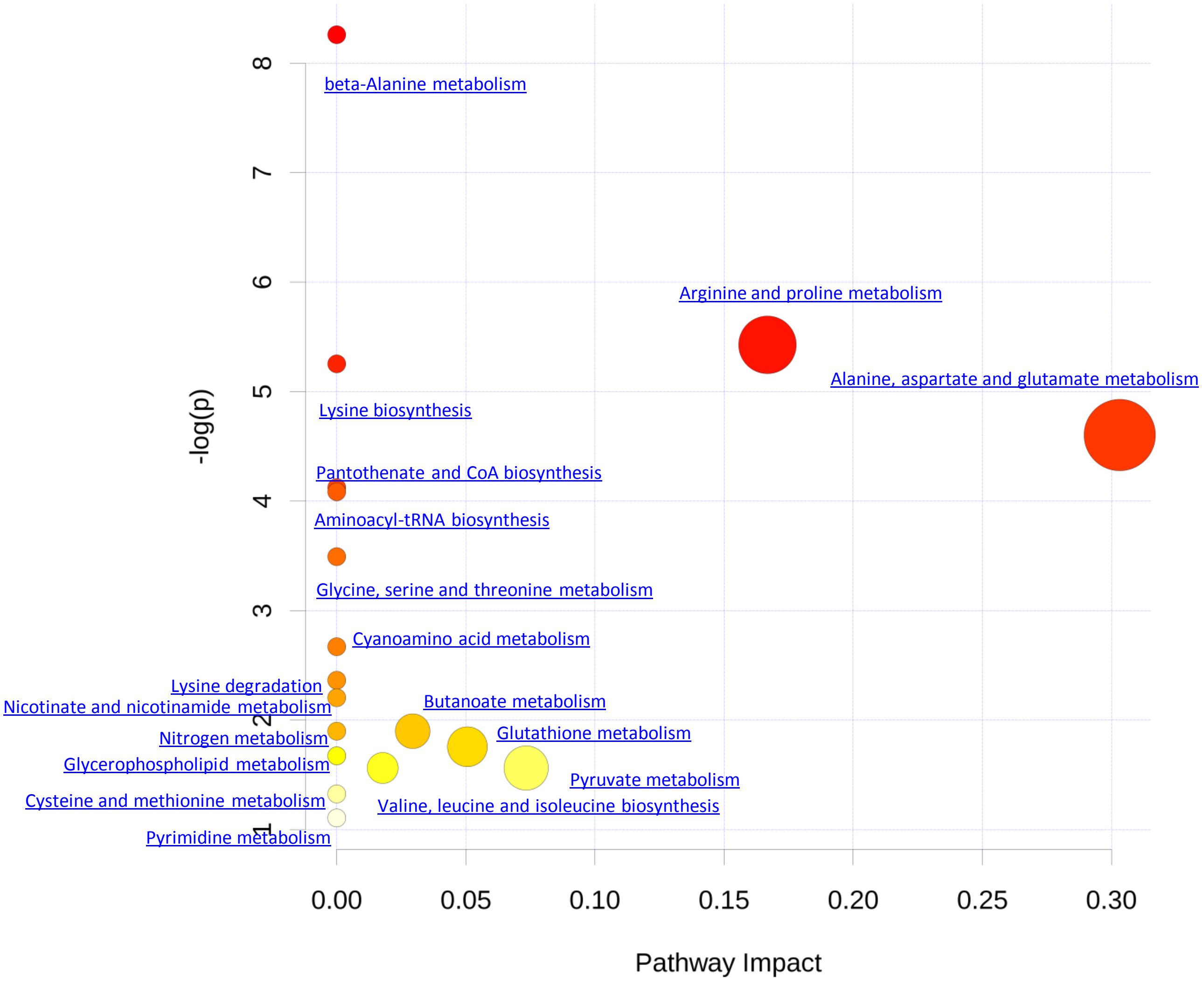

Supplementary Figure 2.2. Network pathway analysis by MetaboAnalyst software showing matched pathways according to p-values from pathway enrichment analysis and pathway impact values from pathway topology analysis based on the identified NMR resonances distinguishing control from the treatment with magainin 2 (125 µg/ml).

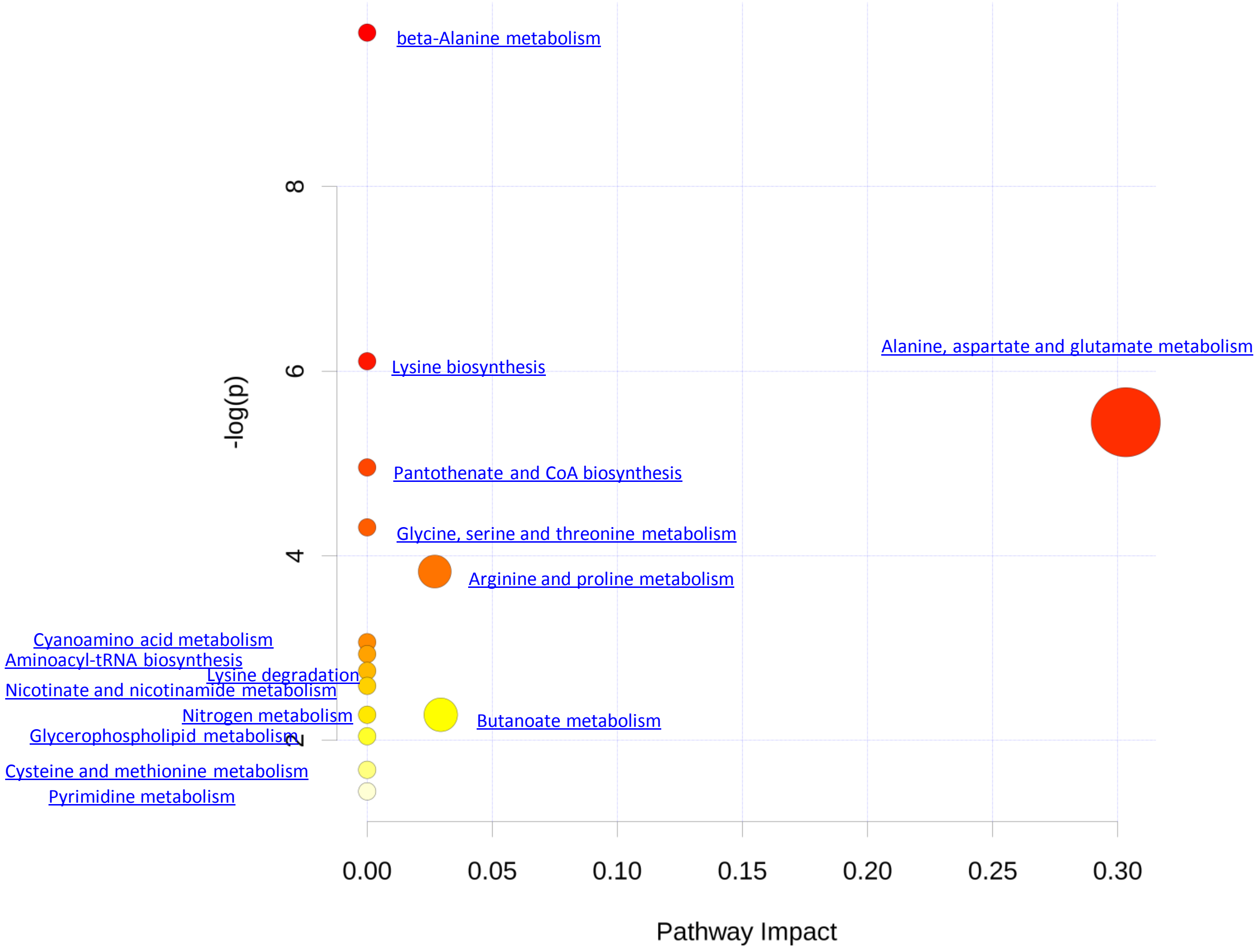

Supplementary Figure 2.3. Network pathway analysis by MetaboAnalyst software showing matched pathways according to p-values from pathway enrichment analysis and pathway impact values from pathway topology analysis based on the identified NMR resonances distinguishing control from the treatment with D-LAK120-AP13 (15.6 µg/ml).

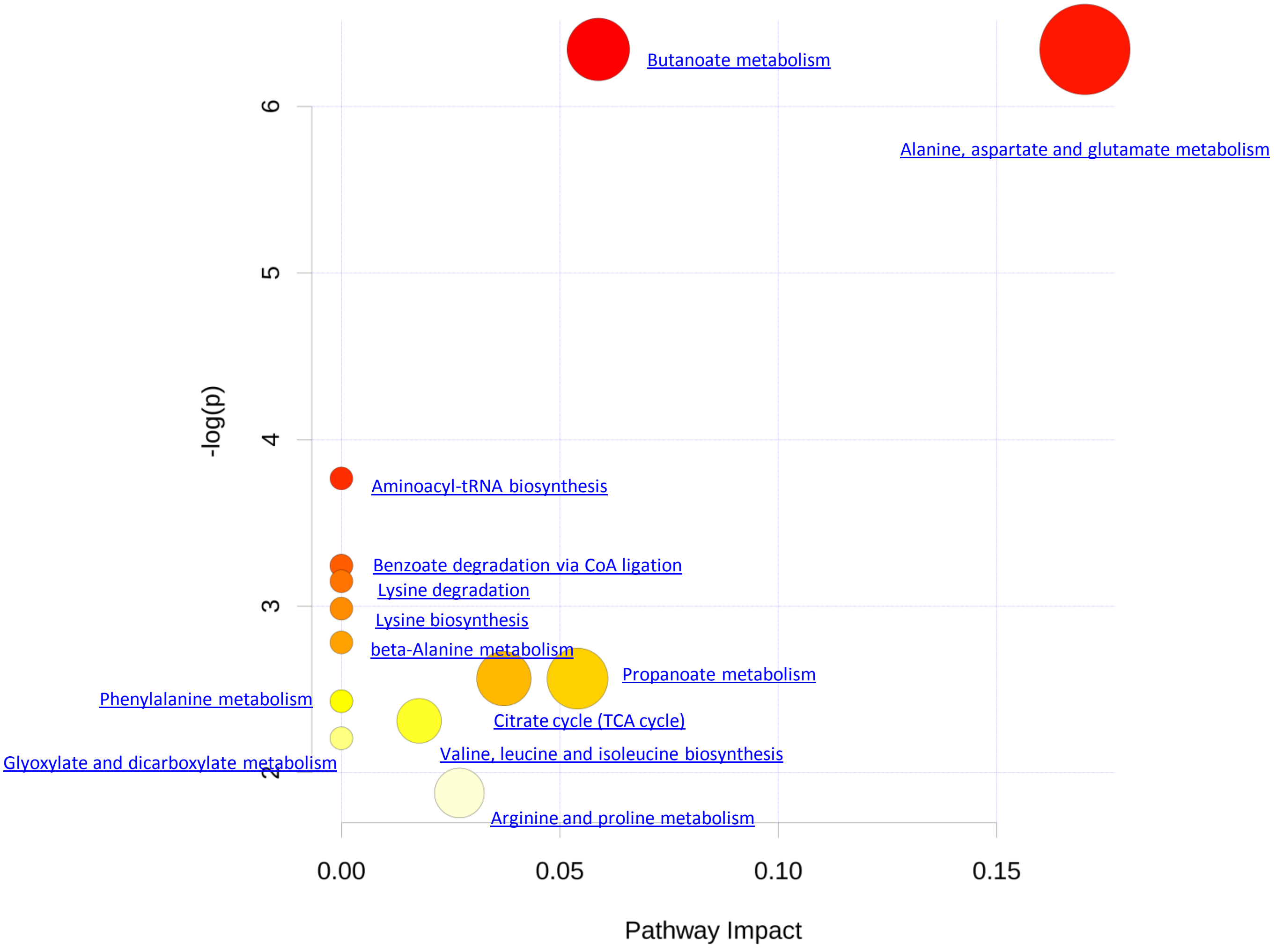

Supplementary Figure 2.4. Network pathway analysis by MetaboAnalyst software showing matched pathways according to p-values from pathway enrichment analysis and pathway impact values from pathway topology analysis based on the identified NMR resonances distinguishing control from the treatment with Buforin II (250 µg/ml).

A

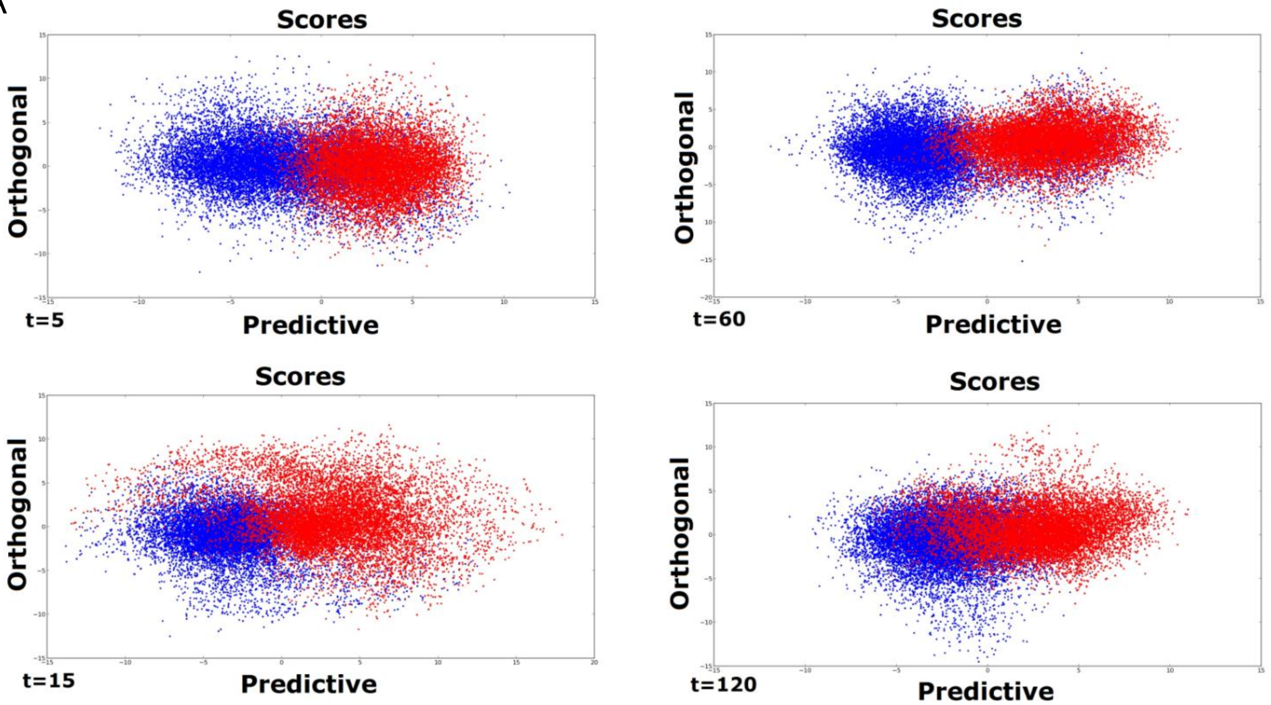

B

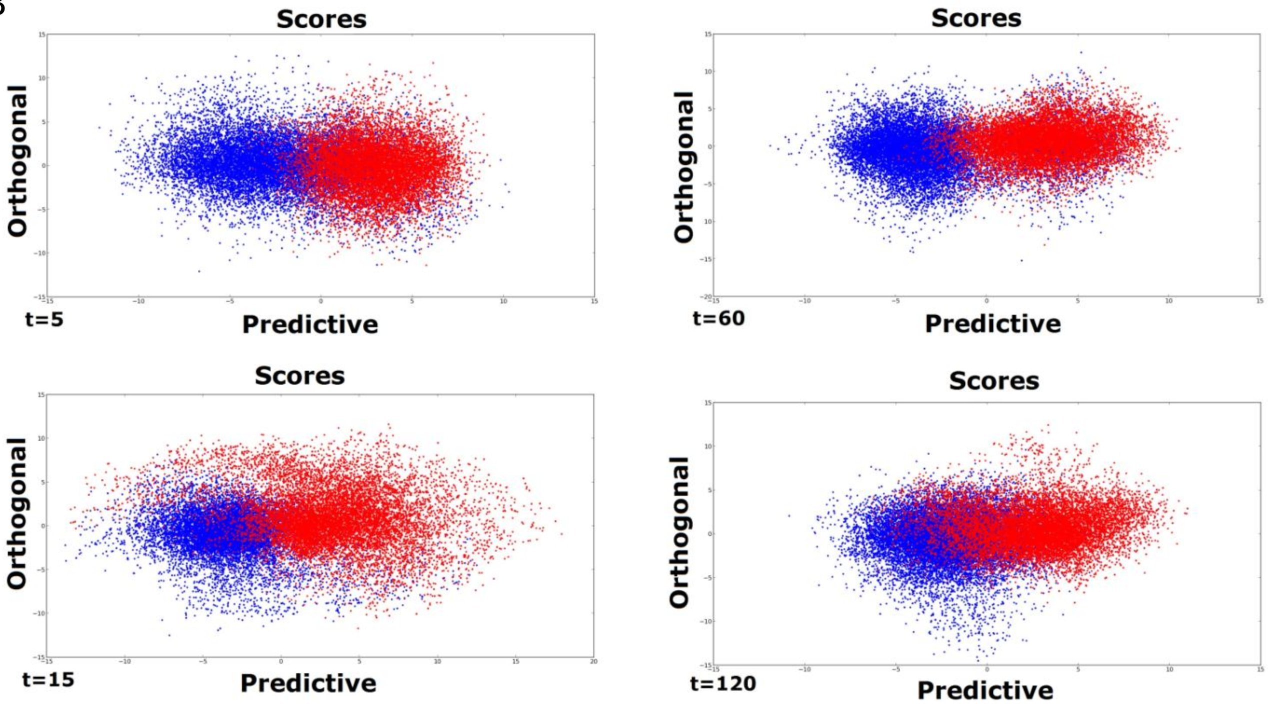

Supplementary Figure 3. Comparison of OPLS-DA scores plot from 2000 cross-validated models for bacteria treated with 125 µg/ml magainin 2 (**A**) or 62.5 µg/ml pleurocidin (**B**)**,** against untreated control at t = 5, t = 15, t = 60, and t = 120 minutes.

Time (mins)	Pleurocidin	Magainin 2
**5**	0.81 (-0.31)	0.51 (-0.25)
**15**	0.86 (-0.27)	0.57 (-0.30)
**60**	0.62 (-0.27)	0.48 (-0.27)
**120**	0.70 (-0.24)	0.49 (-0.25)

Supplementary Table 1. Predictive Q^2 values for OPLS-DA models obtained during the time-course experiment and corresponding to the OPLS-DA scores plots shown in **Supp. Fig. 3**. Q^2 values for cross validation runs with permutated class assignments are given in parentheses.

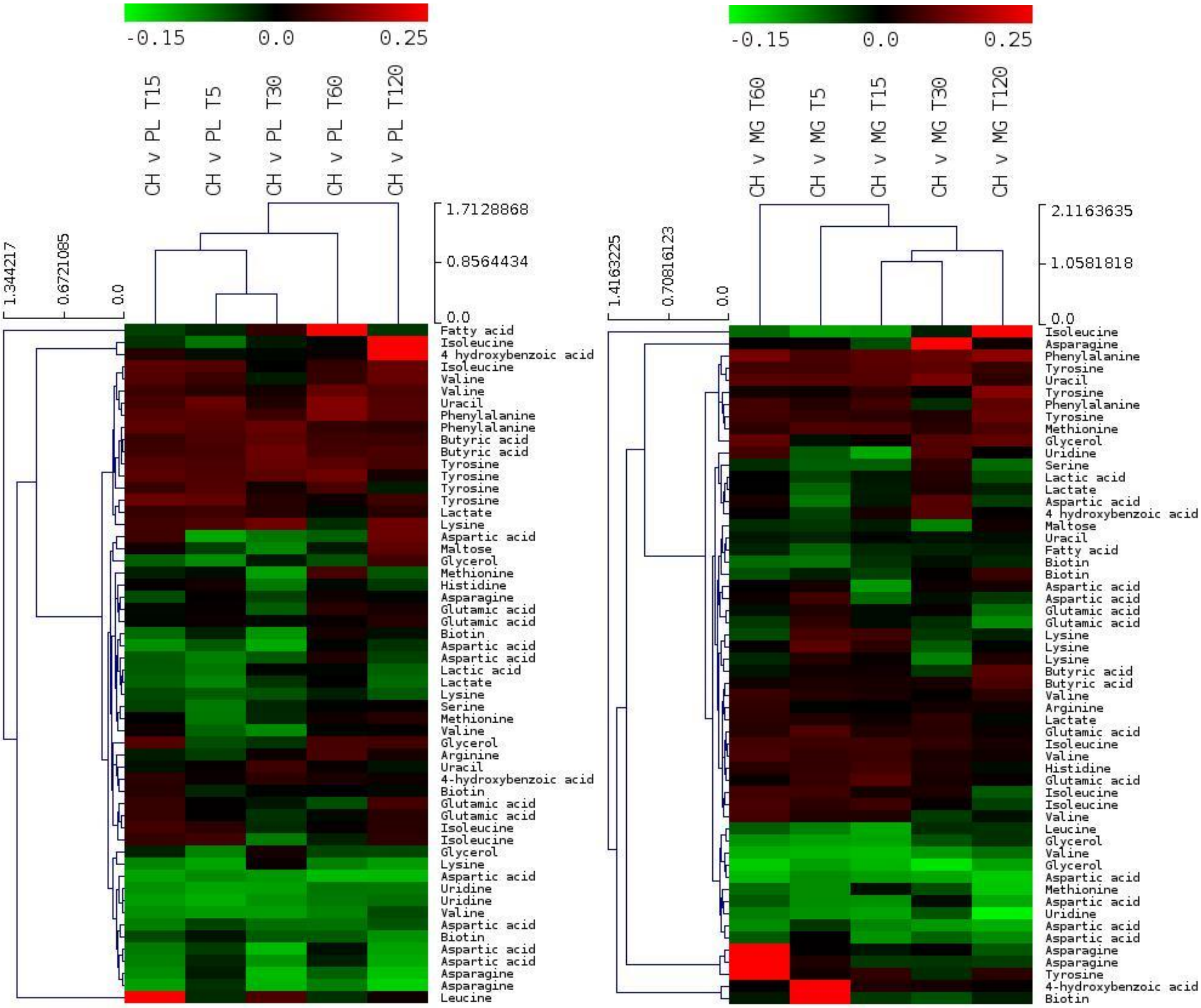

Supplementary Figure 4. Hierarchical cluster analyses of metabolic responses to pleurocidin (**left**) and magainin 2 (**right**) challenge recorded for five different incubation periods. The responses are broadly similar over time but, in particular for pleurocidin, there is a suggestion that a second phase can be detected after c 30 minutes

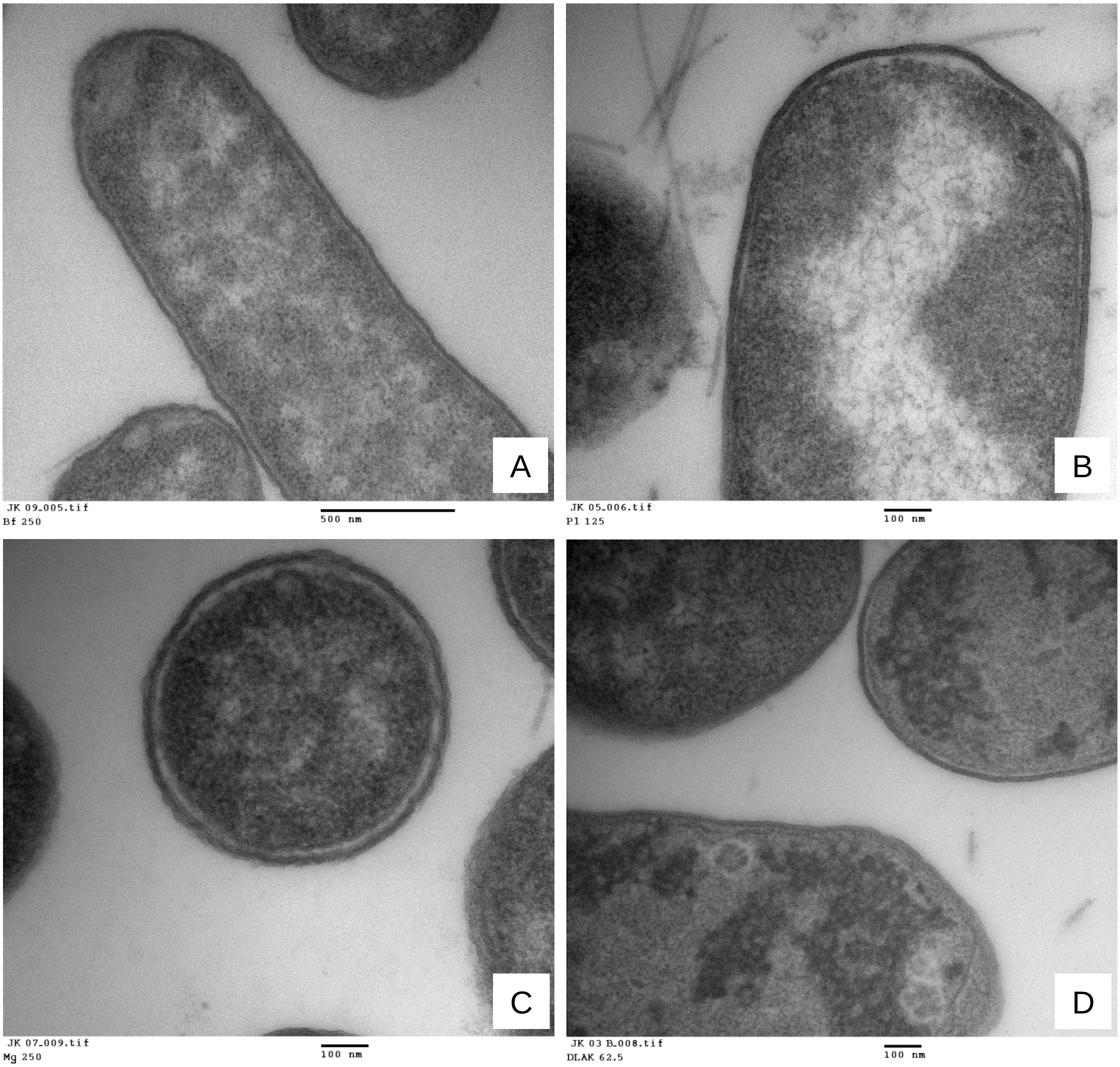

Supplementary Figure 5.1 Transmission electron micrographs AMP challenged *E. coli* NCTC 9001. Bacteria were challenged for 30 minutes with AMPs above the threshold concentration that elicits a bacterial response as determined by the ^{1}H NMR metabolomic study; 250 µg/ml buforin II (**A**), 125 µg/ml pleurocidin (**B**), 250 µg/ml magainin 2 (**C**) and 62.5 µg/ml D-LAK120-AP13 (**D**).

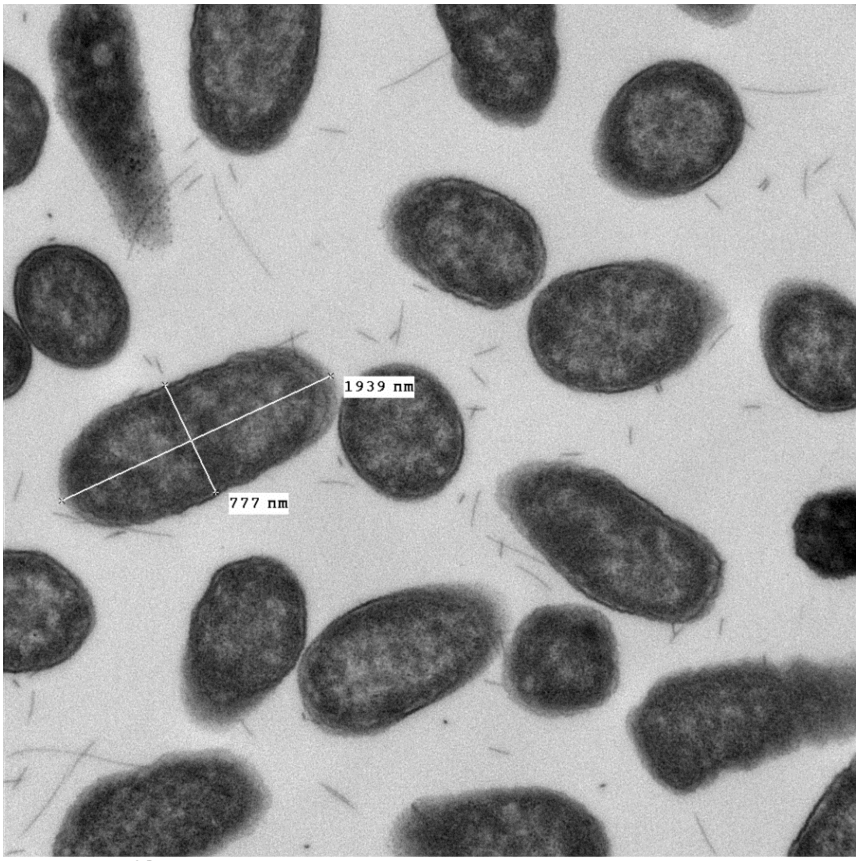

JK 01 B_008.tif
JK01
13:59 05/12/11
Microscopist: JK

500 nm
HV=75.0kV
Direct Mag: 25000x
CUI

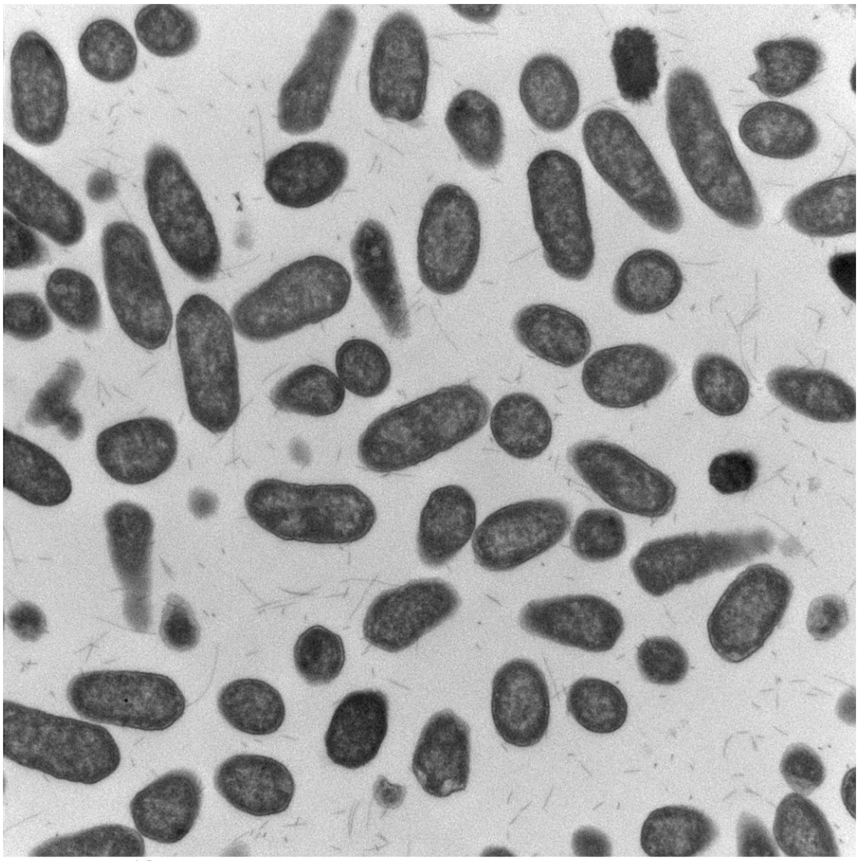

JK 01 B_006.tif
JK01
13:58 05/12/11
Microscopist: JK

2 microns
HV=75.0kV
Direct Mag: 12000x
CUI

Supplementary Figure 5.2 TEMs of *E. coli* NCTC 9001 – control cells

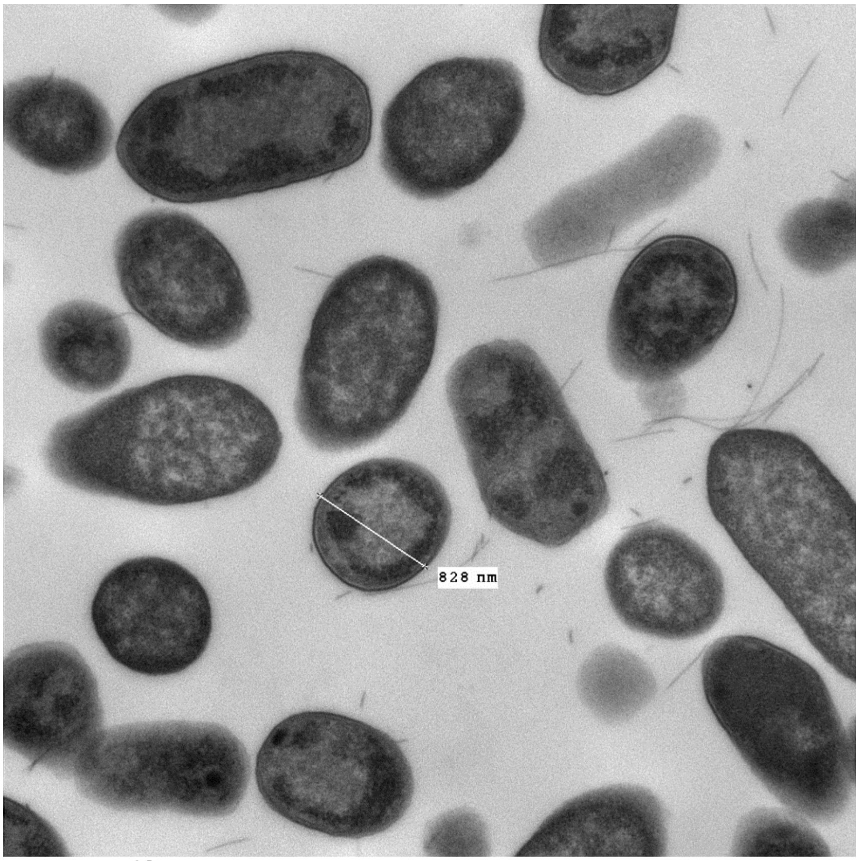

JK 02 B_016.tif
dlak 15.6
14:35 05/12/11
Microscopist: JK

500 nm
HV=75.0kV
Direct Mag: 25000x
CUI

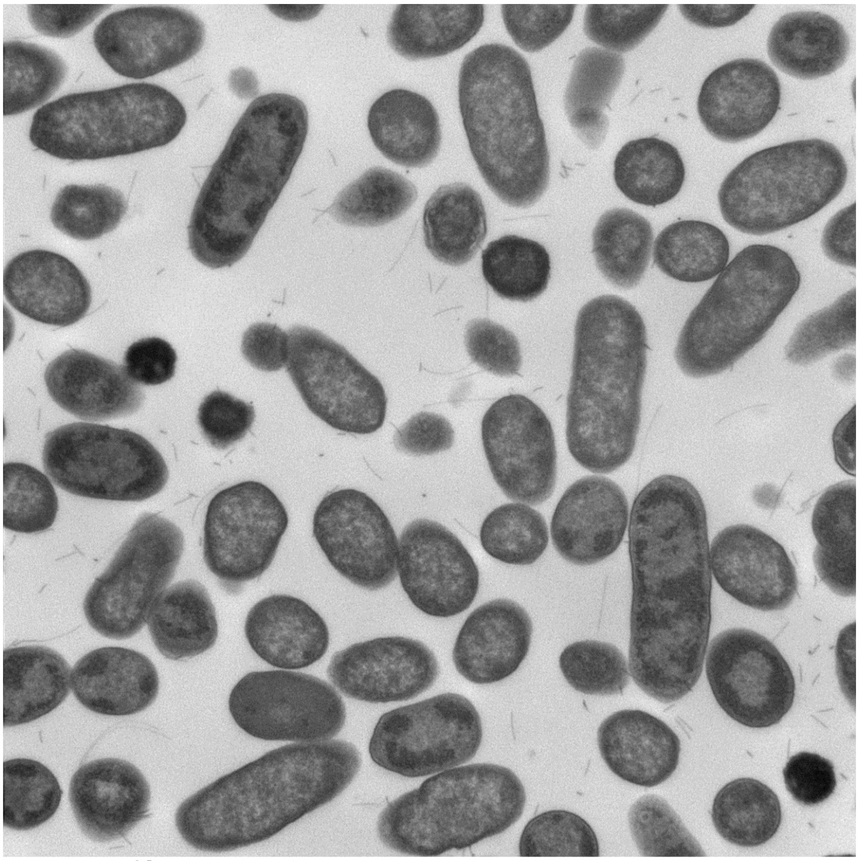

JK 02 B_007.tif
dlak 15.6
14:26 05/12/11
Microscopist: JK

500 nm
HV=75.0kV
Direct Mag: 15000x
CUI

Supplementary Figure 5.3 TEMs of *E. coli* NCTC 9001 challenged with 15.6 µg/ml D-LAK120-AP13

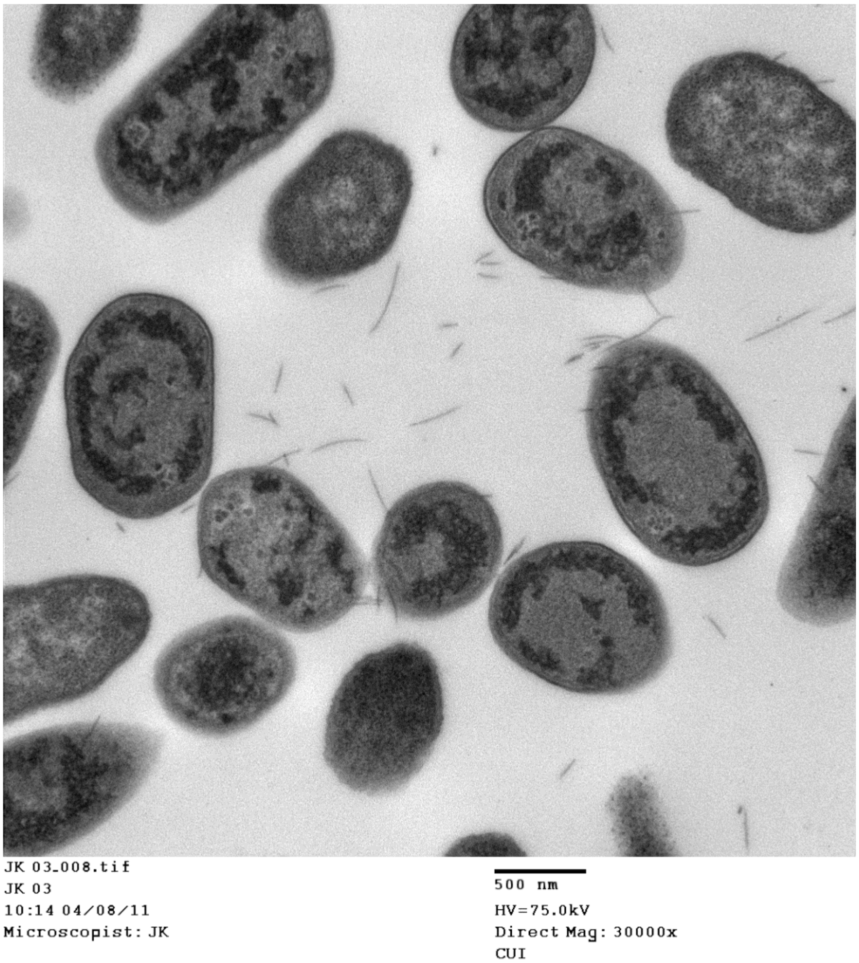

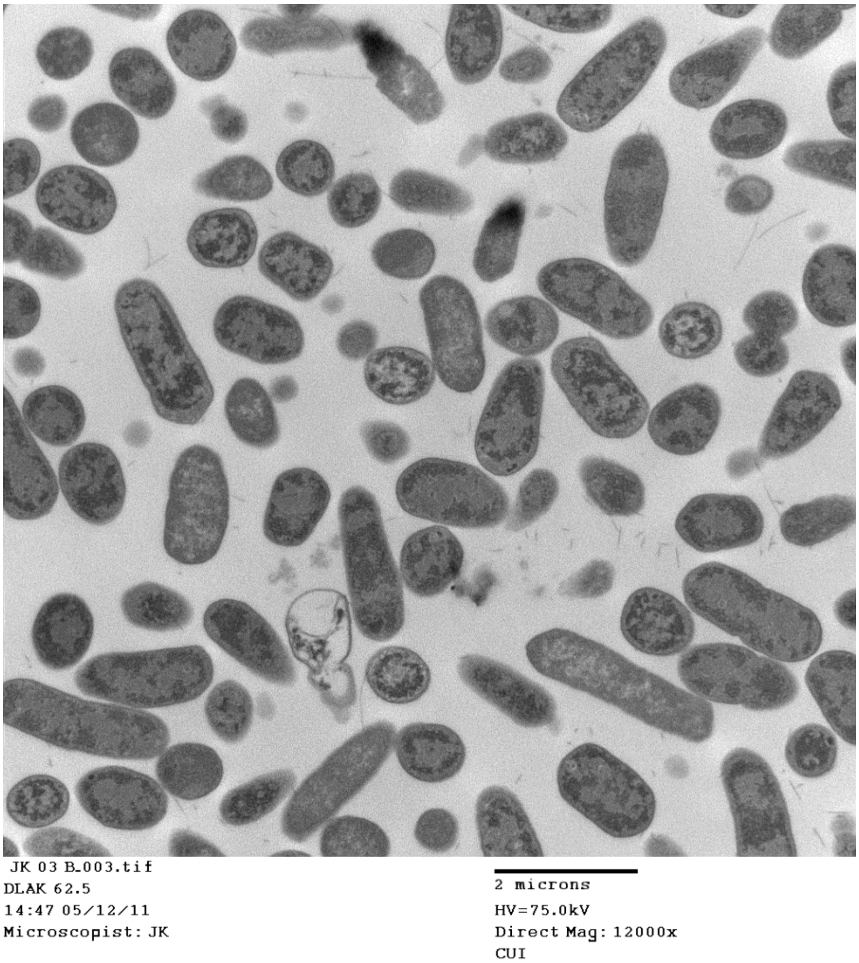

Supplementary Figure 5.4 TEMs of *E. coli* NCTC 9001 challenged with 62.5 µg/ml D-LAK120-AP13

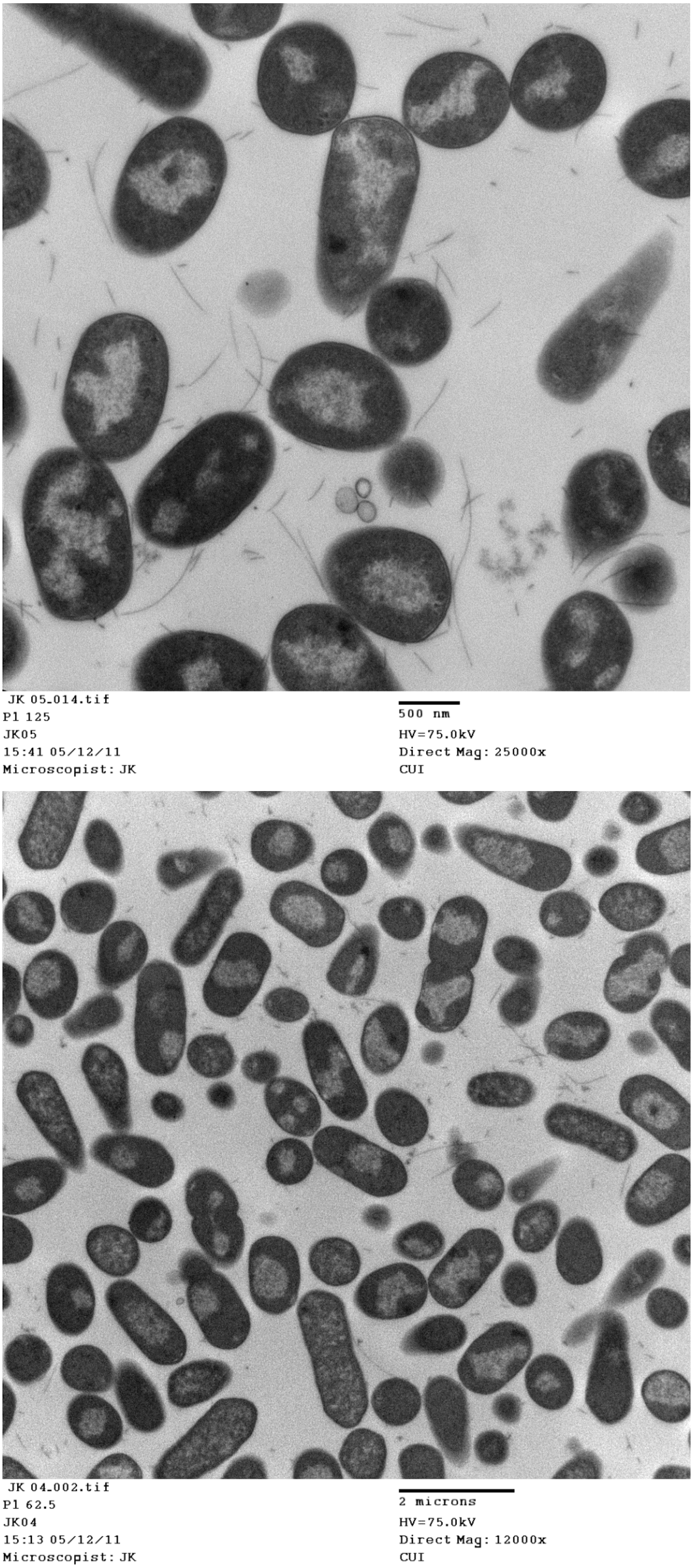

Supplementary Figure 5.5 TEMs of *E. coli* NCTC 9001 challenged with 62.5 µg/ml pleurocidin

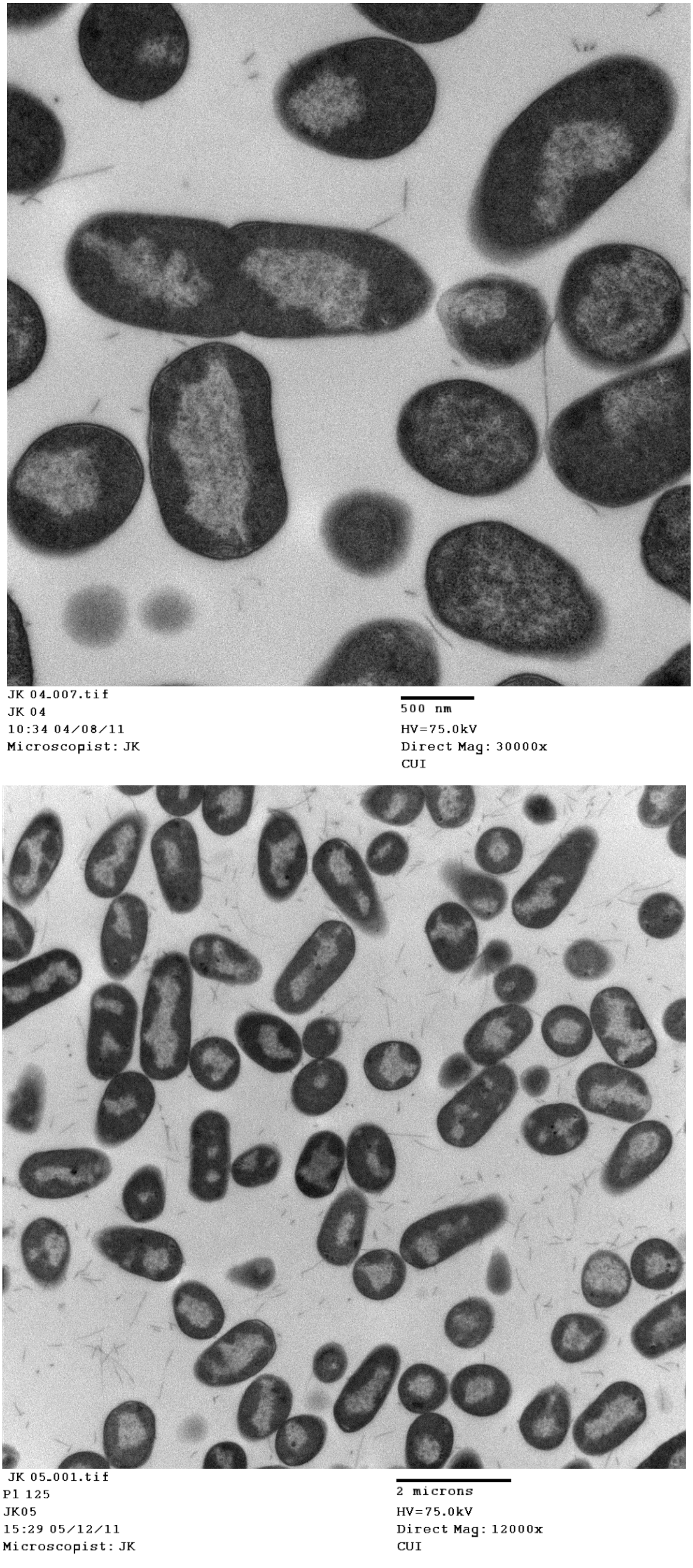

Supplementary Figure 5.6 TEMs of *E. coli* NCTC 9001 challenged with 125 μg/ml pleurocidin

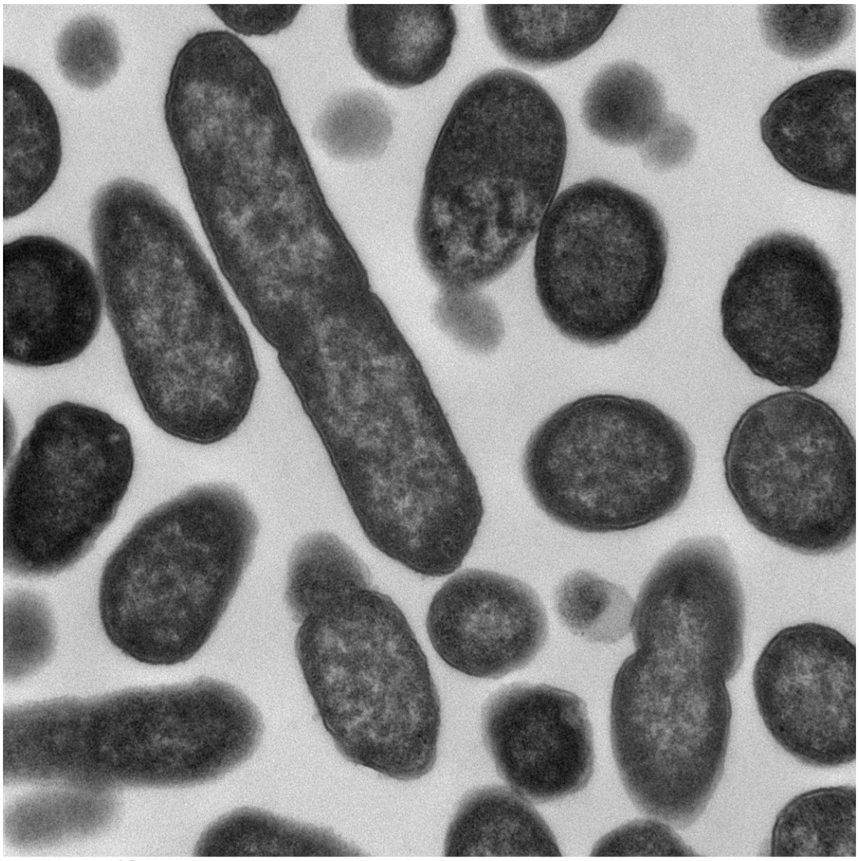

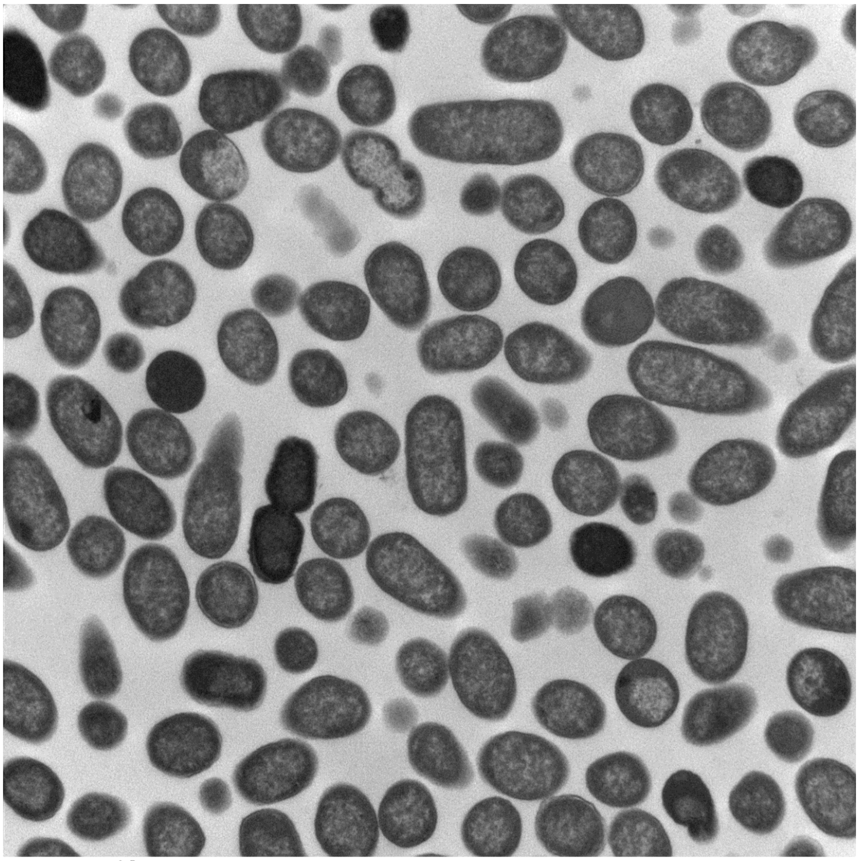

Supplementary Figure 5.7 TEMs of *E. coli* NCTC 9001 challenged with 125 µg/ml magainin 2

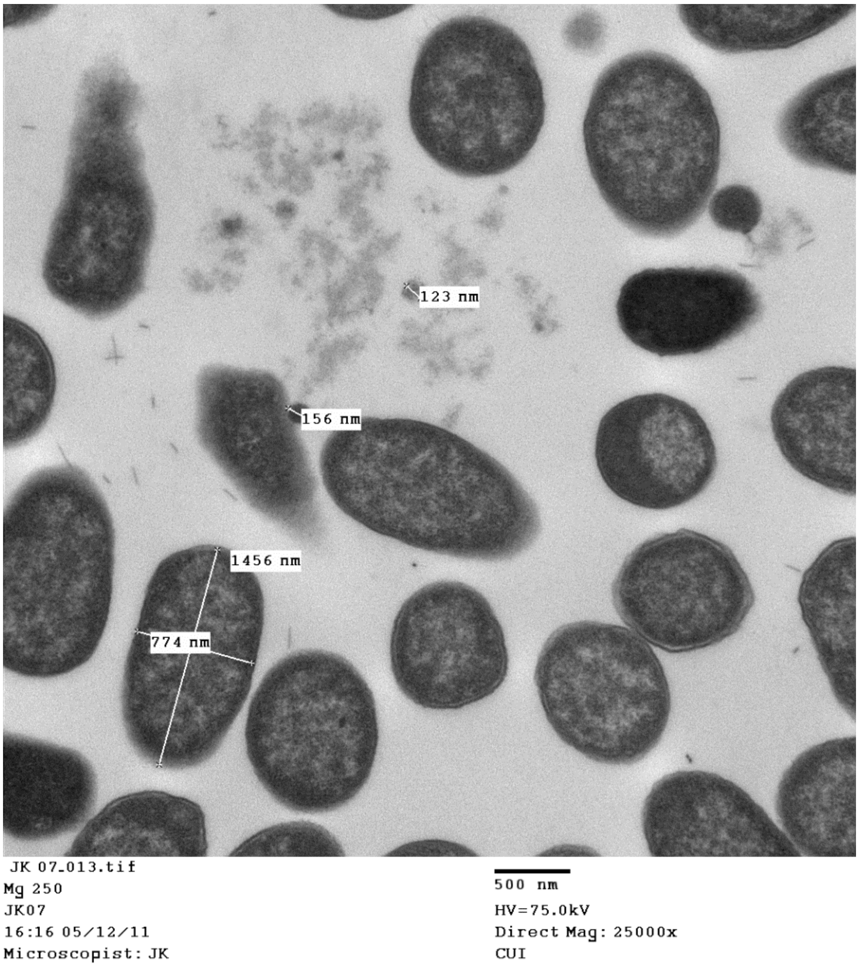

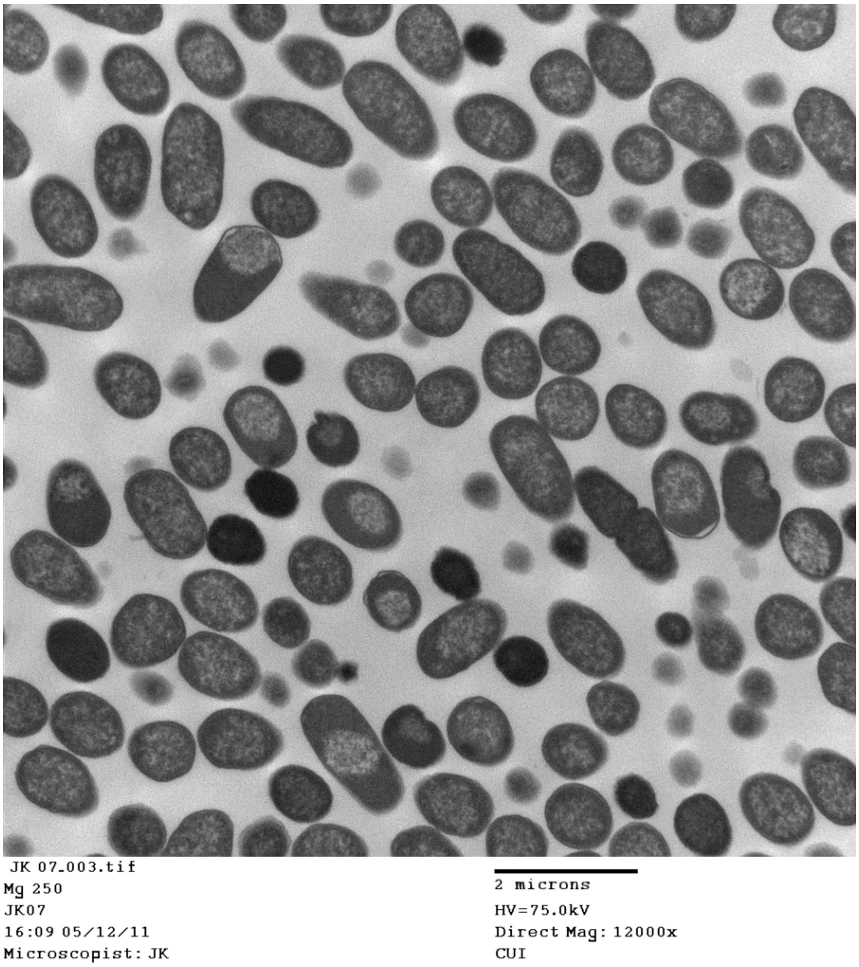

Supplementary Figure 5.8 TEMs of *E. coli* NCTC 9001 challenged with 250 µg/ml magainin 2

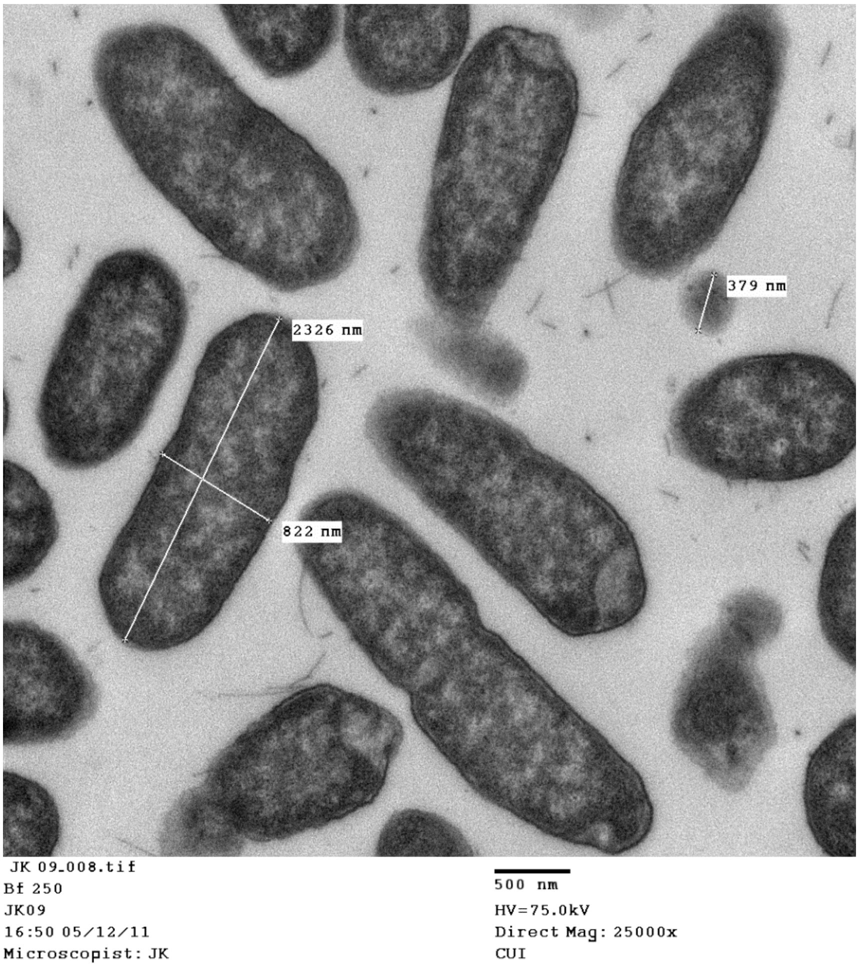

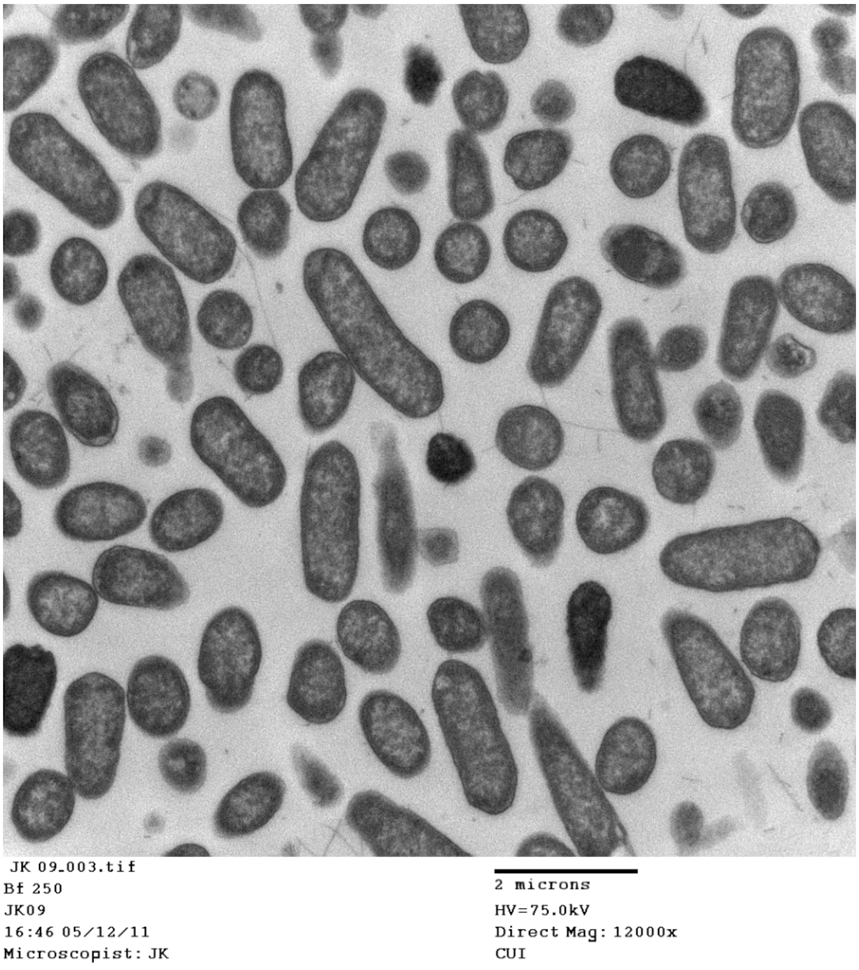

Supplementary Figure 5.9 TEMs of *E. coli* NCTC 9001 challenged with 250 µg/ml buforin II

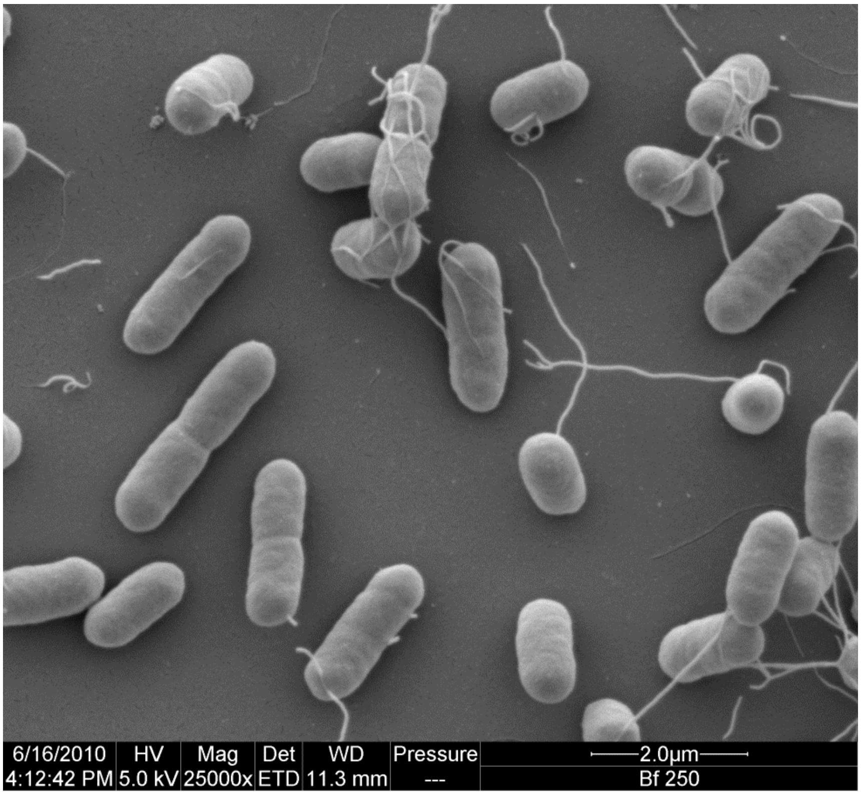

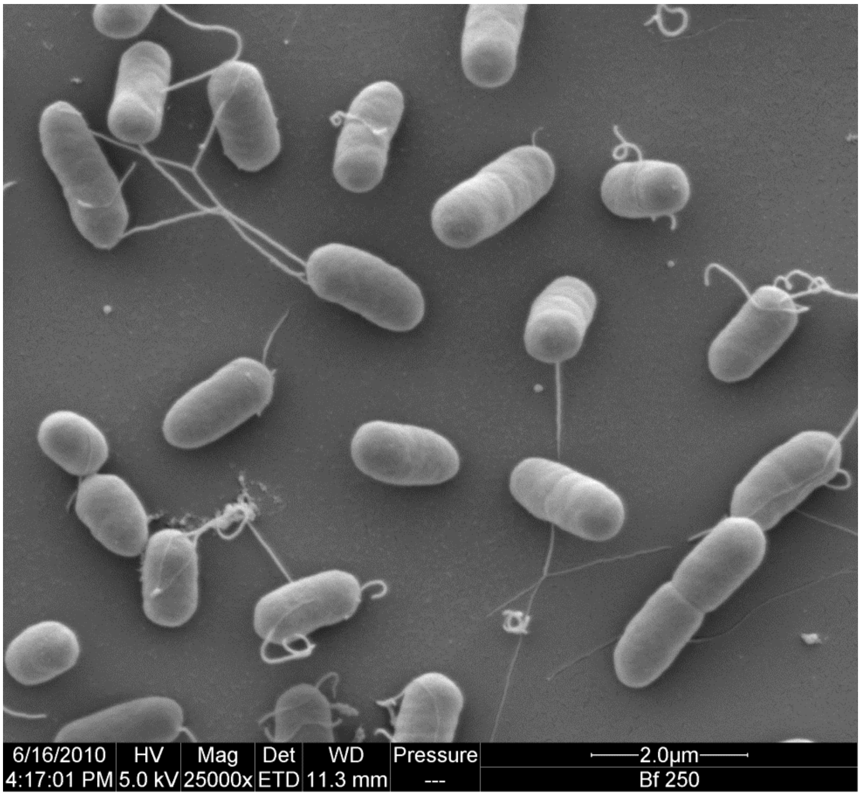

Supplementary Figure 5.10 SEMs of *E. coli* NCTC 9001 challenged with 250 µg/ml buforin II

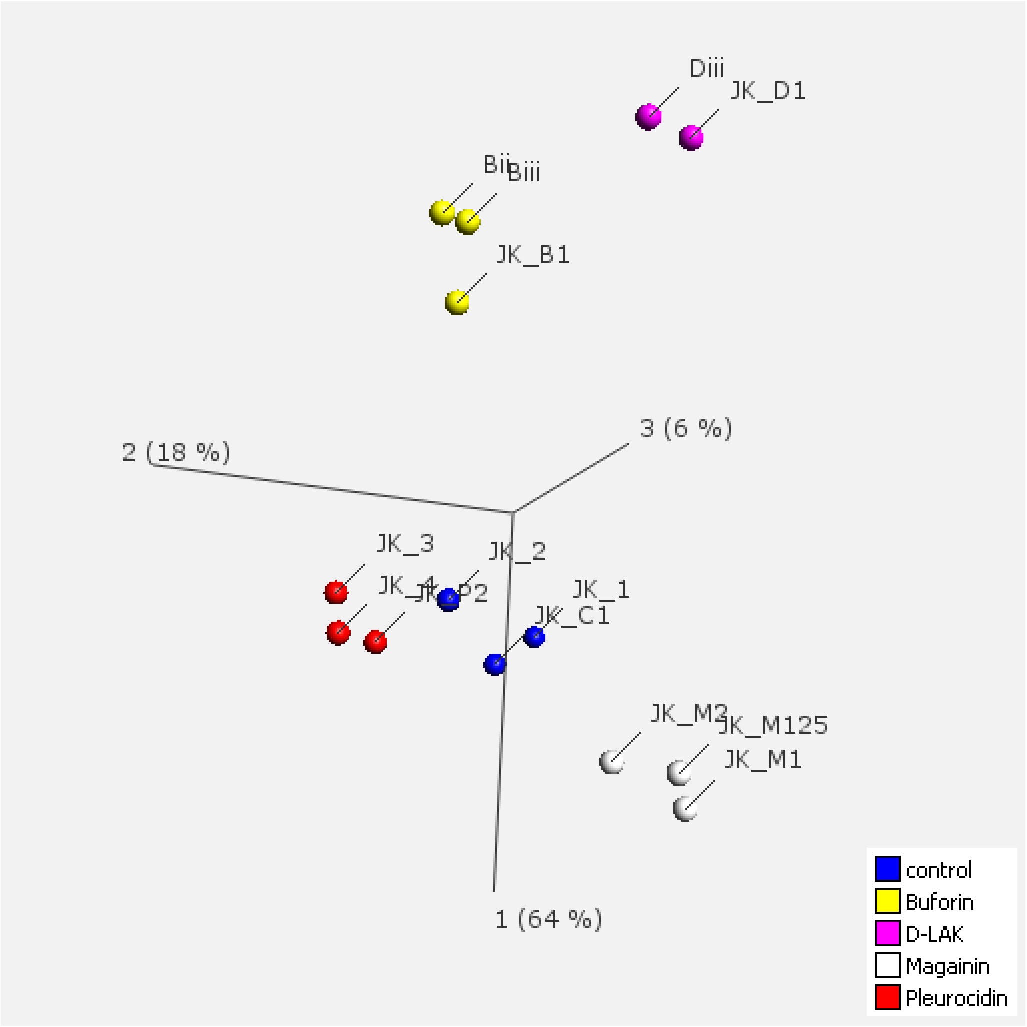

Supplementary Figure 6. Output from Qlucore Omics Explorer showing three dimensional Principal Component Analysis of 20 most differentially expressed genes across all 14 GeneChips for *E. coli* NCTC 9001 as detected by the GeneChip *E. coli* Genome 2.0 Array. Bacteria were challenged for 30 minutes with AMPs at the threshold concentration that elicits a bacterial response as determined by the ^{1}H NMR metabolomic study; 250 µg/ml buforin II, 62.5 µg/ml pleurocidin (**B**), 125 µg/ml magainin 2 (**C**) and 15.6 µg/ml D-LAK120-AP13 (**D**). The axes (1, 2, 3) relate to principal component 1 (PC1) , PC2 and PC3 respectively and indicate how much variance is explained by each of these first three principal components. The plot indicates the reproducibility of the transcript profiling experiment by showing that variance in the 20 most different differentially expressed genes is closely related to the AMP challenge applied.

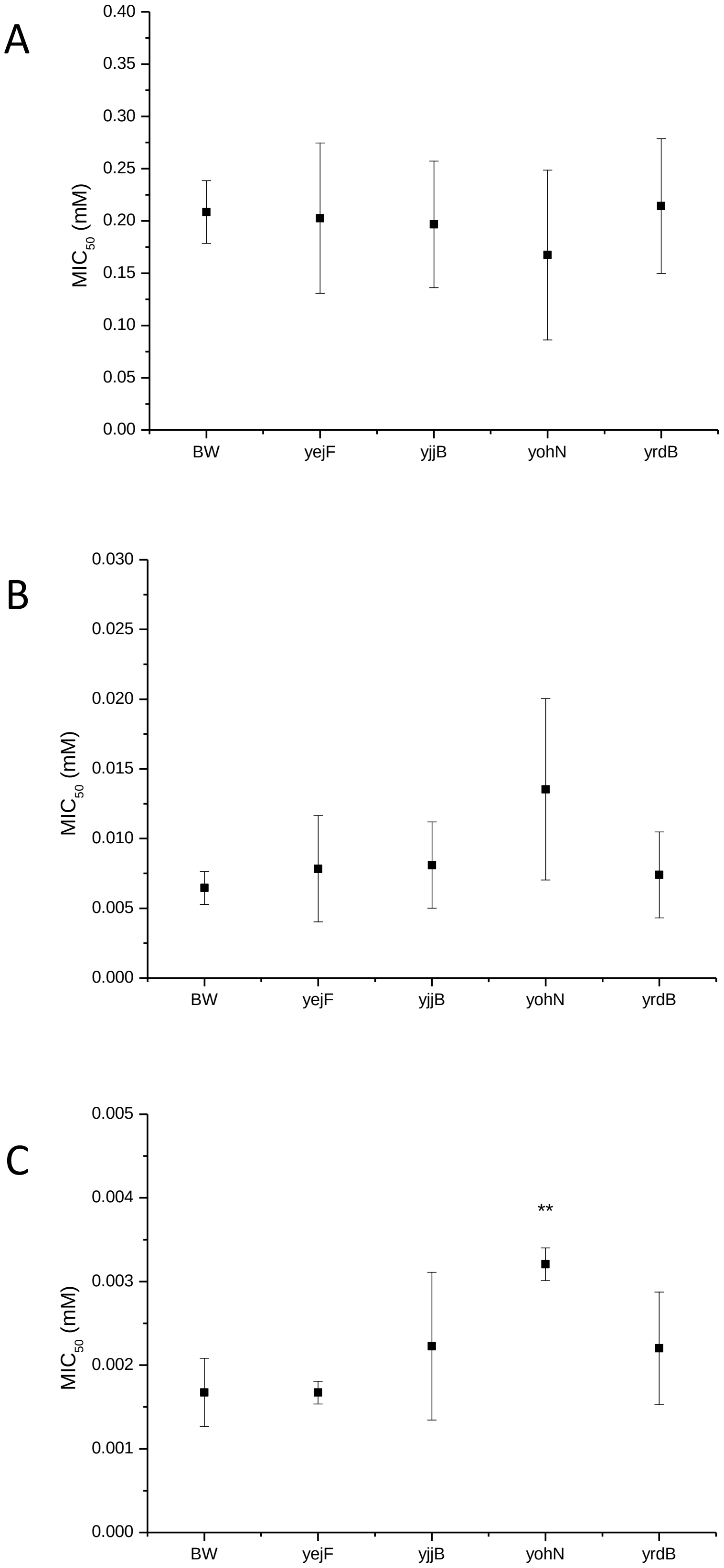

Supplementary Figure 7. Sensitivity of Wild type and four mutants from the Keio collection to different cations: (**A**) $MgCl_2$, (**B**) $NiCl_2$, (**C**) $CoCl_2$. (**) $p \leq 0.05$ relative to BW. Δ*yohN* confers sensitivity to Co^{2+} and possibly Ni^{2+}.

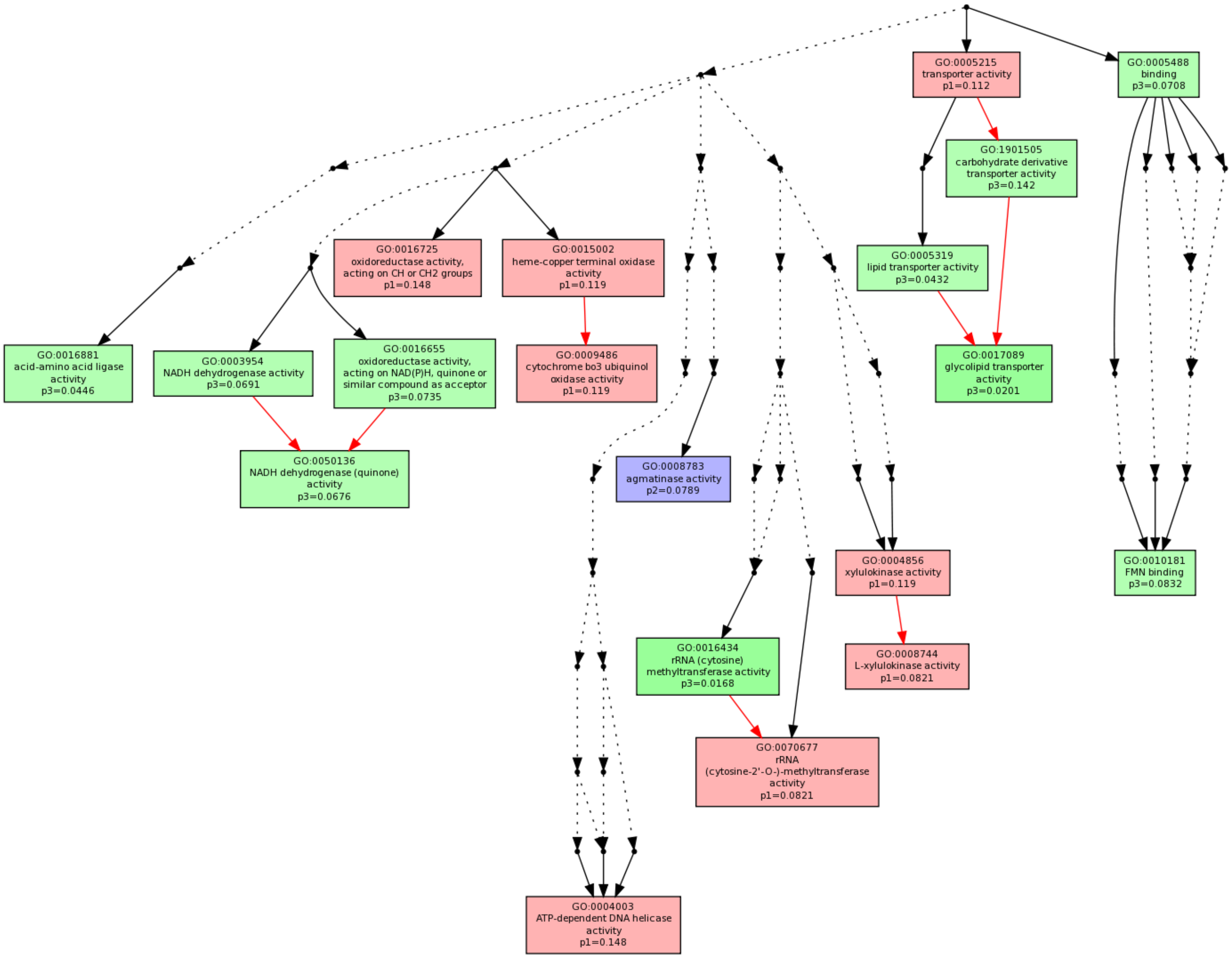

Supplementary Figure 8. Multi GOEAST comparison of molecular function in differentially expressed genes of *E.* coli NCTC 9001 in response to challenge with pleurocidin (red), magainin 2, (blue) and buforin II (green) as detected by the GeneChip® E. coli Genome 2.0 Array. Bacteria were challenged for 30 minutes with AMPs at the threshold concentration that elicits a bacterial response as determined by the ^{1}H NMR metabolomic study; 250 µg/ml buforin II, 62.5 µg/ml pleurocidin and 125 µg/ml magainin 2.

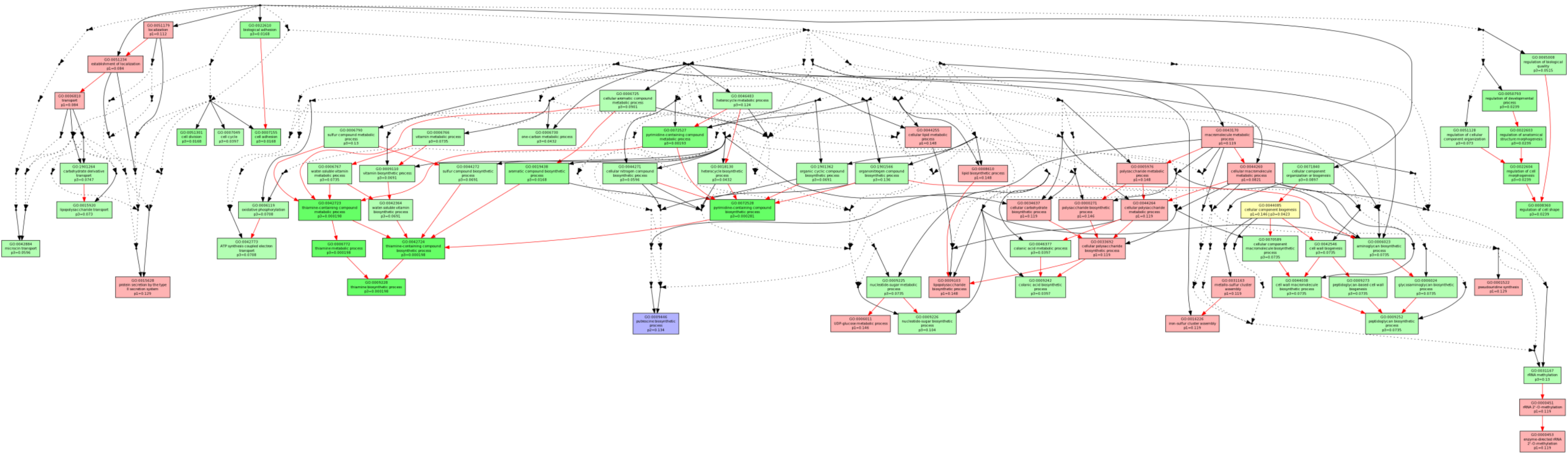

Supplementary Figure 9. Multi GOEAST comparison of biological processes in differentially expressed genes of *E.* coli NCTC 9001 in response to challenge with pleurocidin (red), magainin 2, (blue) and buforin II (green) as detected by the GeneChip® E. coli Genome 2.0 Array. Bacteria were challenged for 30 minutes with AMPs at the threshold concentration that elicits a bacterial response as determined by the ^{1}H NMR metabolomic study; 250 µg/ml buforin II, 62.5 µg/ml pleurocidin and 125 µg/ml magainin 2.

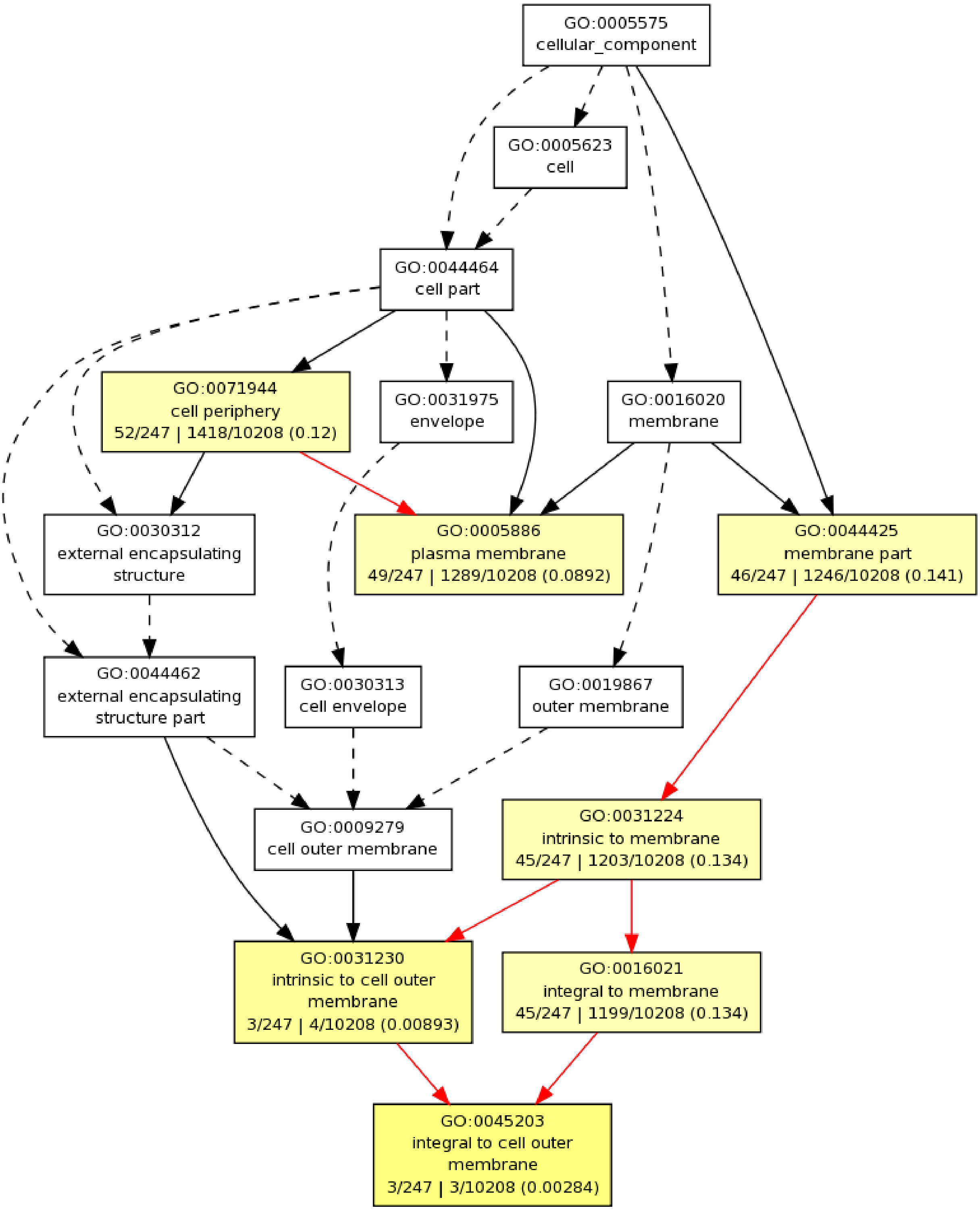

Supplementary Figure 10. GOEAST analysis of cellular component in differentially expressed genes of *E.* coli NCTC 9001 in response to challenge with magainin 2 as detected by the GeneChip® E. coli Genome 2.0 Array. Bacteria were challenged with 125 µg/ml magainin 2; the threshold concentration that elicits a bacterial response as determined by the ^{1}H NMR metabolomic study.

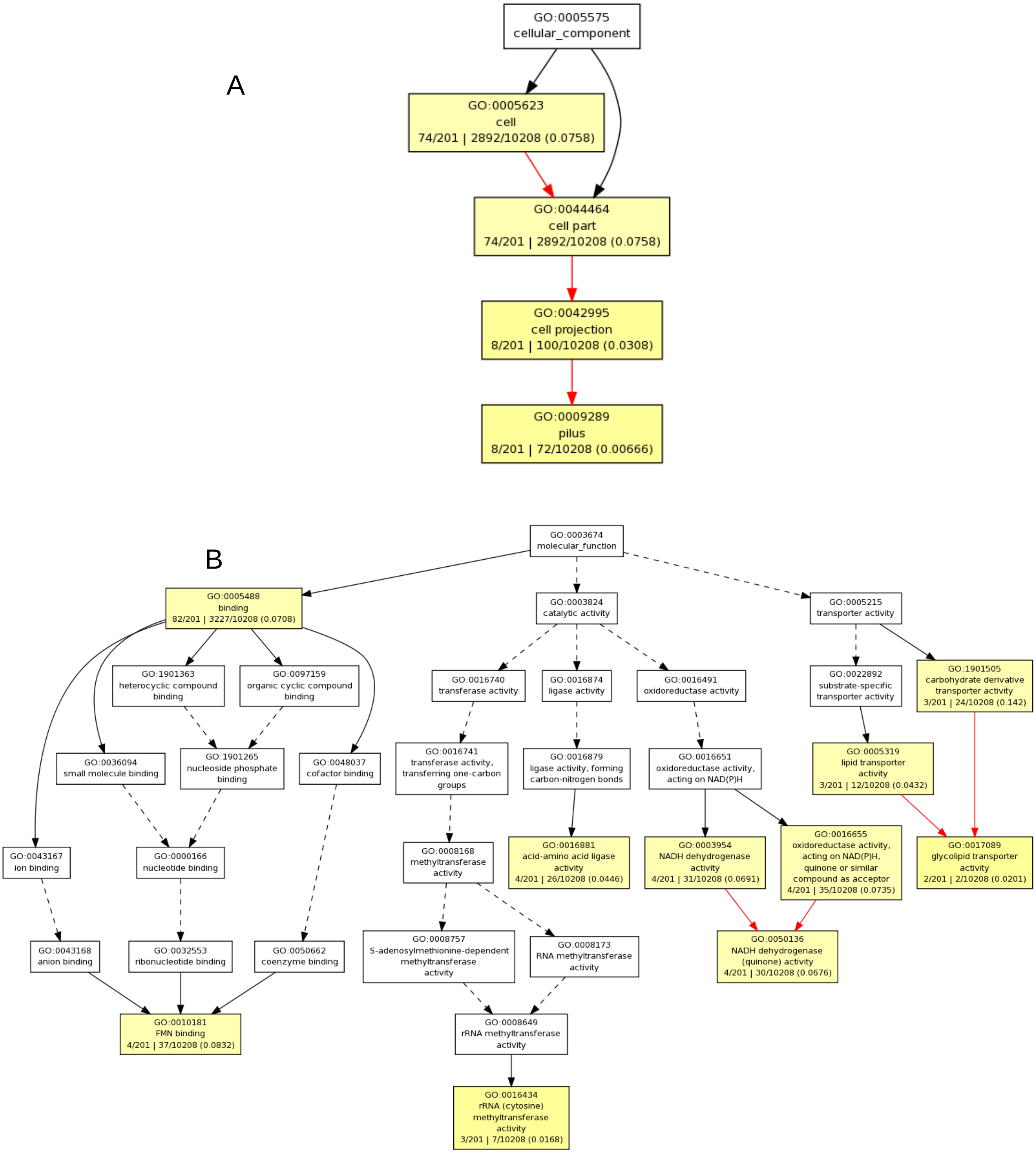

Supplementary Figure 11 GOEAST analysis of cellular component (**A**) and molecular function (**B**) in differentially expressed genes of *E.* coli NCTC 9001 in response to challenge with buforin II as detected by the GeneChip® E. coli Genome 2.0 Array. Bacteria were challenged with 250 µg/ml buforin II; the threshold concentration that elicits a bacterial response as determined by the ^{1}H NMR metabolomic study. Note the concentration of genes in cellular component GO terms "cell" or "cell part" and in molecular function GO:0005488 "binding".

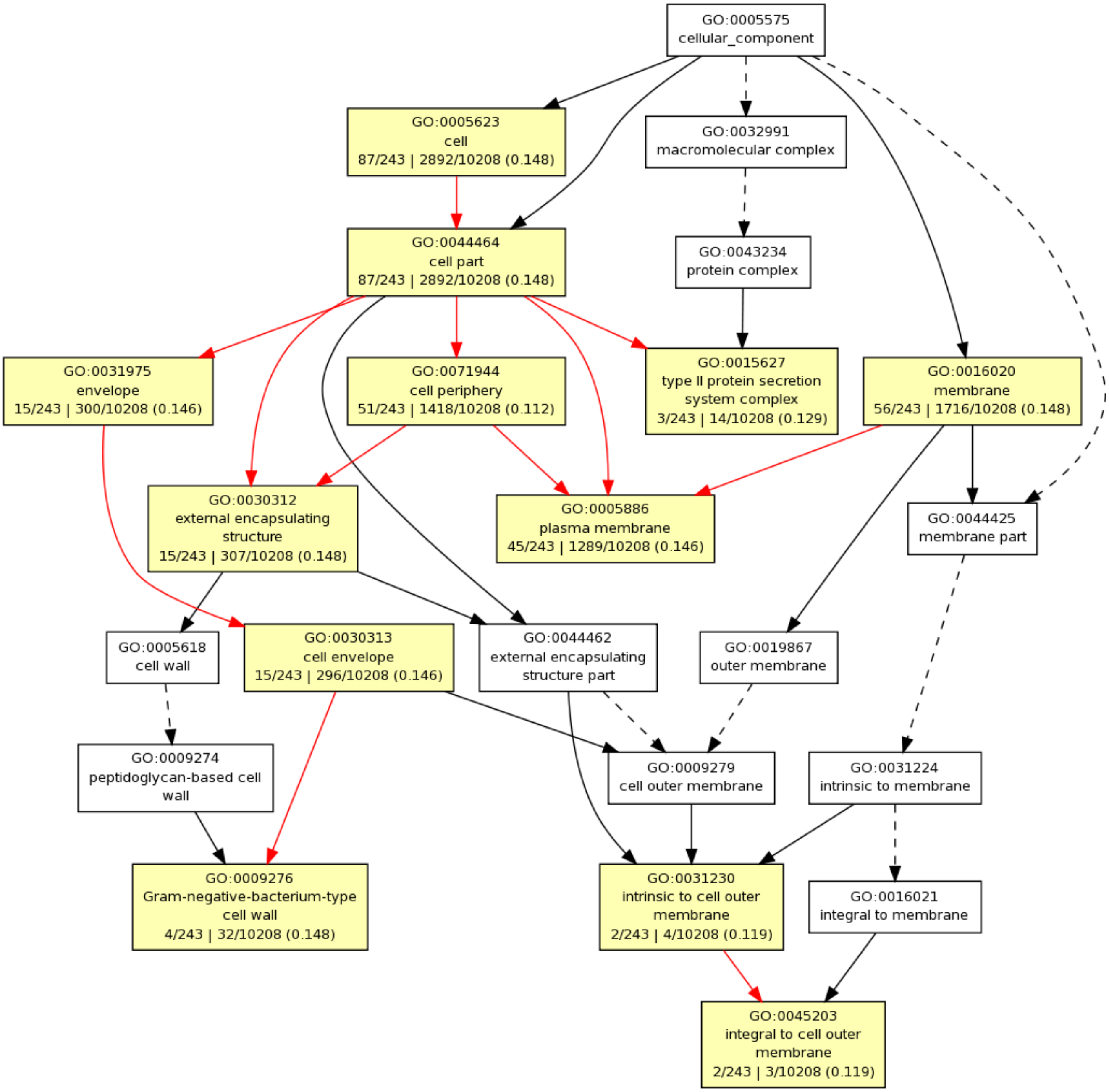

Supplementary Figure 12. GOEAST analysis of cellular component in differentially expressed genes of *E.* coli NCTC 9001 in response to challenge with pleurocidin as detected by the GeneChip® E. coli Genome 2.0 Array. Bacteria were challenged with 62.5 µg/ml pleurocidin; the threshold concentration that elicits a bacterial response as determined by the ^{1}H NMR metabolomic study. Note the distribution of genes between GO terms “cell”/”cell part”, “cell periphery” and “membrane”/”plasma membrane”.

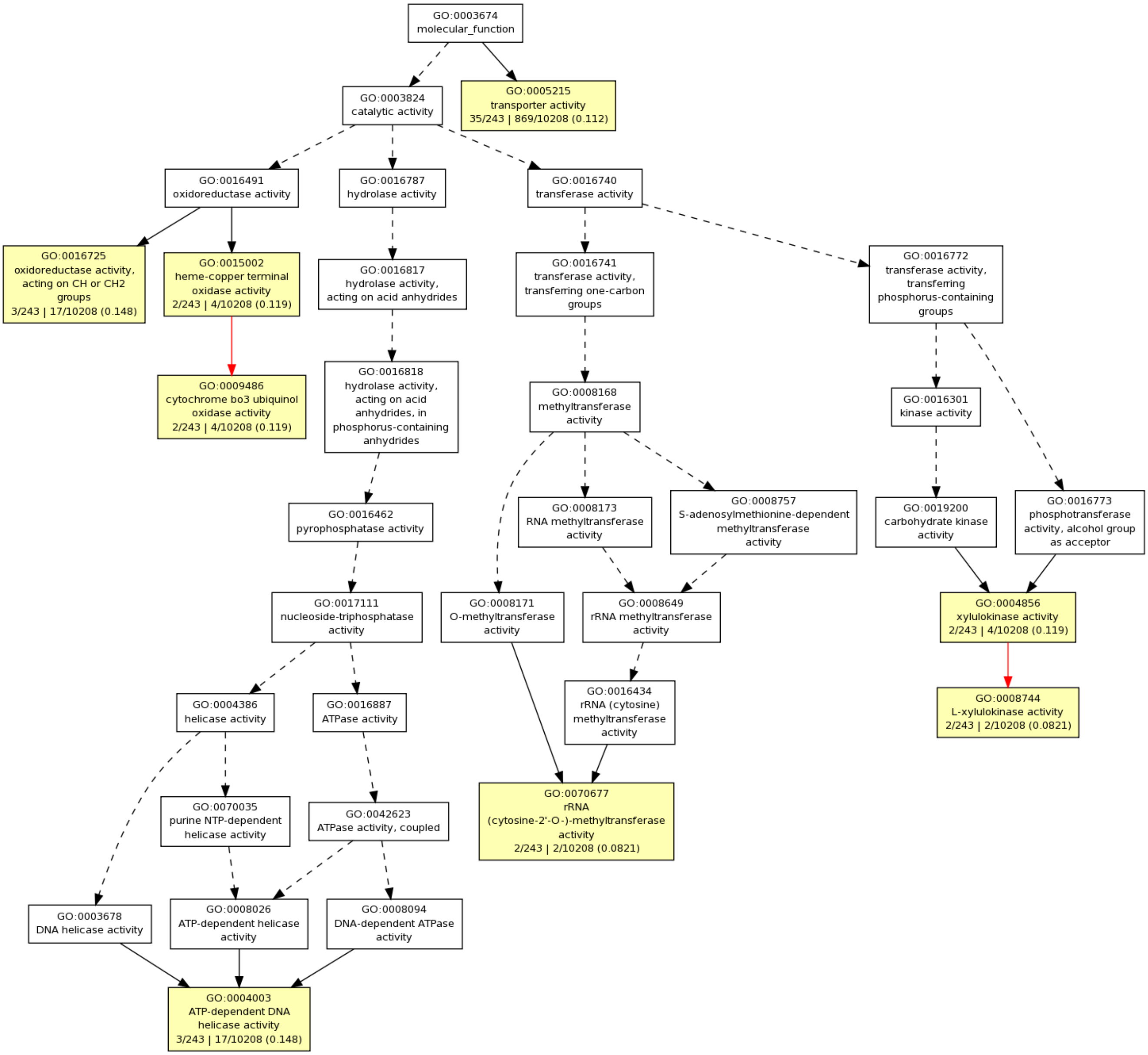

Supplementary Figure 13. GOEAST analysis of molecular function in differentially expressed genes of *E.* coli NCTC 9001 in response to challenge with pleurocidin as detected by the GeneChip® E. coli Genome 2.0 Array. Bacteria were challenged with 62.5 µg/ml pleurocidin; the threshold concentration that elicits a bacterial response as determined by the ^{1}H NMR metabolomic study. Note the high number of genes corresponding to GO:0005215 transporter activity.
